# Exploring regulatory roles of putrescine-doped zinc oxide nanoentities on ethylene signaling, redox imbalance, and programmed cell death in drought-stressed rice (*Oryza sativa* L.) seedlings

**DOI:** 10.3389/fpls.2025.1630837

**Published:** 2025-08-19

**Authors:** Abir Das, Tibor Janda, Sudipta Kumar Sil, Malay Kumar Adak

**Affiliations:** ^1^ Plant Physiology and Molecular Biology Research Unit, Department of Botany, University of Kalyani, Kalyani, West Bengal, India; ^2^ Department of Plant Physiology and Metabolomics, Agricultural Institute, HUN-REN Centre for Agricultural Research, Martonvásár, Hungary; ^3^ Department of Botany, University of Gour Banga, Malda, West Bengal, India

**Keywords:** drought stress, chloroplast, endogenous polyamines, nanoparticle, putrescine, rice

## Abstract

We investigated the synergistic effects of putrescine-doped zinc oxide nanoparticles (PUT-nZnO) on drought-stressed rice seedlings. Our results demonstrate that PUT-nZnO enhances drought stress (DS) tolerance by improving redox balance, chloroplast integrity, and polyamine (PA) metabolism, offering a novel nano-biotechnological approach for crop resilience. Fourteen-day-old seedlings of rice (*Oryza sativa* L. cv. Swarna Sub1) were treated with PUT by foliar spray, singly and in combination with PUT-nZnO under 12% polyethylene glycol (PEG)-induced DS. Growth attributes, thermo-imaging, chloroplast ultrastructure, PA and ethylene signaling, relative cell death, redox metabolism, and nuclear lysis were the major parameters used to evaluate stress mitigation. DS initially caused a 48% decrease in relative water content, which was recovered to 126% under PUT-nZnO treatment. PUT-nZnO directly improved membrane integrity, reduced DNA loss, restored ion homeostasis via ATP hydrolysis, and supported cellular conformity and viability. These effects reduced DS-induced oxidative signaling through enhanced antioxidation. Oxidative stress under DS was mitigated, as indicated by a 41% reduction in H_2_O_2_ in the DS+PUT-nZnO treatment. Distribution of PAs and the activity of PA-oxidizing enzymes induced energy transfer within the chloroplast and reactive oxygen species (ROS) generation to activate enzymatic pathways. The mechanism for DS tolerance is indicated by nZnO through securing osmotic turgidity and mineral nutrient support, complemented synergistically by the antioxidation capacity of PUT. This study presents a promising biocompatible strategy for improving drought tolerance in rice during the early growth stage via the combined application of PUT and nZnO.

## 1 Introduction

In rice cultivation, drought stress (DS) is (1) a common factor that limits growth and productivity by compromising various physiological responses. Rice (*Oryza sativa* L.), being a semi-aquatic plant, often struggles to maintain proper tissue moisture, which impedes seed germination, seedling establishment, reproductive development, and leads to early senescence (Lim et al., 2023). A soil-moisture regime below the permanent wilting point induces DS, which restricts nearly every cellular response necessary for adequate crop development. Under DS, failure of seed germination results in hindered seedling growth, reduced acquisition and partitioning of dry matter, disrupted allocation of photoassimilates, impaired stomatal regulation, and increased susceptibility to lodging (Ullah and Farooq, 2022; Jan et al., 2025).

Therefore, for successful crop cultivation, the selection of rice genotypes capable of withstanding DS becomes a pivotal issue in ensuring satisfactory productivity. Moreover, adjusting cellular responses to DS by maintaining tissue moisture can sustain plant growth. This can be achieved through the exogenous application of growth regulators, nutrient supplements, elicitors, and genetic modifications. Among various chemical elicitors for mitigating water stress, certain low-molecular-weight organic compounds have proven particularly effective, with PAs being among the most important (Jangra et al., 2023).

Chemically, PAs are polycationic, aliphatic, straight-chain residues with an alkaline pH. The predominant residues, such as putrescine (PUT) and its derivatives spermidine (SPD) and spermine (SPM), are abundant in plants (Islam et al., 2020). PAs play vital roles in cellular functions, including cell division, tissue differentiation, and the regulation of transcriptomes and proteomes. Key physiological functions include membrane stabilization, ion and solute transport, osmotic adjustment, CO_2_ assimilation, allocation in biometabolites, and overall growth modulation.

The activity of PAs is primarily focused on regulating stress tolerance, particularly under water and oxidative stress conditions (Yadav et al., 2025). Their multifaceted roles in cellular responses to drought stress (DS) often involve the amplification of signals mediated through reactive oxygen species (ROS), including their generation and decomposition. In maintaining cellular redox homeostasis, PAs play a significant role in modulating hydrogen peroxide (H_2_O_2_)—a ROS—within the threshold cellular concentration. Thus, maintaining redox homeostasis emerges as an alternative mechanism to cope with DS, where oxidative degeneration of biomolecules presents a major vulnerability.

The established roles of PAs in mitigating drought and oxidative stress are attributed to their polycationic interactions with negatively charged residues such as membrane phospholipids, amino acids, glycerophosphates and esters, and nucleic acid motifs (Hassan et al., 2020; Kumar et al., 2021). Additionally, PA metabolism, which releases specific ROS such as H_2_O_2_, can activate antioxidative cascades under DS. The exogenous application of PAs, either directly or in formulated mixtures, has been effectively adapted to modulate stress tolerance, yielding positive impacts on *Triticum* species, including enhanced carbon assimilation (Doneva et al., 2021; Pál et al., 2025).

The combination of PAs and other growth regulators has also been well documented in the induction of genes associated with water stress tolerance (Gholizadeh et al., 2024; Zargar et al., 2025). Drought stress (DS)-induced lodging sensitivity in rice seedlings has been linked to the alteration of growth regulators, such as ethylene, under semi-dried soil or intermittent drought conditions. Significant accumulation of ethylene and its catabolism, resulting in reactive oxygen species (ROS) generation, accelerates early senescence in rice leaf sheaths (Yang et al., 2017; Qiao et al., 2024). This phenomenon is evident in rice genotypes containing specific quantitative trait loci (QTL) that express genes related to ethylene metabolism and sensitivity under DS (Das et al., 2024b). Regulating ethylene metabolism and understanding its impact on cellular functions are crucial criteria in selecting rice genotypes for DS tolerance.

Advances in nanobiotechnology are also gaining importance in mitigating water stress in crops. Improved formulations or combinations of nanoparticles (NPs) and PAs have shown success (Ahmadi-Nouraldinvand et al., 2023; Rehman et al., 2024; Yang et al., 2024). Genotypes that overproduce PAs can be modulated through the exogenous application of NPs, providing simultaneous tolerance to osmotic deficit and oxidative redox imbalance. Genetic variations in PA metabolism, complemented by effective NP applications, have positively influenced the expression of photosynthetic and antioxidative genes under osmotic stress (Alsherif et al., 2022; Al-Elwany et al., 2022; Tyagi et al., 2023). These observations encouraged the investigation of NP-mediated induction of PA metabolism as a regulator of drought sensitivity in crops. A nanocomposite was synthesized in which nano zinc oxide (nZnO) was doped with PUT, following appropriate opto-physical characterizations (Gupta et al., 2024).

With regard to cellular integrity, which often becomes vulnerable under stress, PAs may support its recovery. Still, the exact molecular targets modulated by PAs are not clearly understood. While PAs can bind negatively charged residues through electrophilic interactions, such binding may also interfere with ion transport in cells—an aspect that remains to be clarified. These knowledge gaps present challenges in optimizing the interaction between nanoentities and target molecules. PUT-nZnO was applied to rice seedlings via foliar sprays at regular intervals under polyethylene glycol (PEG)-induced DS. Although the literature on DS in rice cultivation and its mitigation through chemical treatments is well developed, a research gap persists. Specifically, the interaction between PUT-nZnO and cellular signaling—particularly regarding ethylene sensitivity, gene expression related to cellular organelle integrity, PA catabolism-derived H_2_O_2_, and nuclear disorganization—remains underexplored. Therefore, we hypothesized that PUT-nZnO would serve as an effective and biocompatible treatment that helps plants regain osmotic turgidity and recover from DS-induced oxidative stress, thereby sustaining cellular integrity and function.

In essence, this study aimed to investigate the impacts of PUT-nZnO and to unravel the specific roles of physiological and cellular mechanisms involved in drought tolerance in rice seedlings. Key areas of focus included ethylene signaling, PA metabolism, oxidative redox balance, chloroplast ultrastructural changes, DNA damage, and physiological responses. The findings of this study, supported by scientific validation, are expected to provide insights into potential interactions between nanoparticles (NPs) and PAs, thereby contributing to improved drought tolerance in rice.

## 2 Materials and methods

### 2.1 Synthesis and characterization of nanoentities

The synthesis of nZnO followed the method established in our previous research on biofabrication using *Arthrospira platensis* (Das et al., 2024a). Briefly, 0.1% (w/v) synthesized nZnO was mixed with double-distilled water (ddH_2_O) to form a homogeneous solution. Next, 1 g of PUT (Sigma, Germany) was dissolved in 500 mL of 0.1% (w/v) nZnO solution, and the solution was stirred until the PUT was fully dissolved (~40 min) at 35°C. Afterward, the PUT-nZnO solution was treated with 1% (v/v) Tween-20 (Sigma, Germany) to facilitate encapsulation of the bioactive compound. Finally, the nanoentities were sonicated (1 h) to achieve uniform dispersion, and characterized through different opto-physical methodology.

### 2.2 Plant material and treatments

Rice (*Oryza sativa* L. cv. Swarna Sub1) seeds were surface sterilized with 0.2% (w/v) mercuric chloride for 6 min and kept at 35°C for 2 days in the dark to promote sprouting. Once germinated, the seedlings were transferred to Hewitt nutrient solution (pH 5.8 ± 0.2) for 7 days to acclimate under laboratory conditions [temperature: 28–30°C (day, 14 h) and 25–28°C (night, 10 h); relative humidity: 75%–80%; irradiance: 1000–1200 µE m^-^² s^-^¹], according to our established protocol with minor modifications (Das et al., 2024b).

A solution of PUT (50 mg L^-^¹) was mixed with 0.1% nZnO to prepare the PUT-nZnO nanoentities. An aliquot of 0.1% PUT-nZnO was sprayed onto the leaves of 7-day-old seedlings (two-leaf stage) for two consecutive days. After treatment, the plants were acclimated for 1 day under standard laboratory conditions and then transferred to 12% (w/v) PEG-6000 (Sigma, Germany) prepared in 100 mL of Hewitt solution in identical 250 mL glass containers to induce drought stress (DS) (Das et al., 2024a; Jan et al., 2022). Control (CK) plants were sprayed with the same volume of ddH_2_O.

The experiment included four treatment groups: control (CK), PEG-induced drought stress (DS), foliar application of PUT (50 mg L^-^¹), and PUT-nZnO (0.1%). To prevent nutrient depletion, treatment solutions were refreshed every third day. All treatments were performed in triplicate, arranged in a completely randomized block design with 15 seedlings per replicate, maintaining uniform plant density. Growth parameters were measured after 14 days. For biochemical and molecular analyses, samples were flash-frozen in liquid nitrogen and stored at −80°C.

### 2.3 Measurements of morphological attributes

Morphological attributes including shoot and root length, leaf length, width and area, and the ratio of fresh weight (FW) to dry weight (DW) were measured according to Niu et al. (2022).

### 2.4 Determination of relative water content and relative membrane permeability

After 14 days of drought exposure, leaves from each treatment were collected and promptly transferred to the laboratory for RWC analysis. First, the FW of the leaves was measured. The leaves were then submerged in ddH_2_O for 24 h under dark conditions to estimate the turgid weight (TW). Subsequently, the leaves were placed in a hot air oven at 80°C to record the DW and calculate the RWC using the following formula:.


RWC=[FW-DW/TW-DW]×100

Briefly, detached leaf slices (1 cm) were placed in Falcon tubes containing 10 mL of ddH_2_O, vibrated for 30 s, and the initial electrical conductivity (EC_0_) was recorded. The tubes were then refrigerated for 24 h, after which EC_1_ was measured. Following autoclaving, the same samples were analyzed for EC_2_, and the relative membrane permeability (RMP) was calculated.


$$\text{RMP}\left(\%\right)=\left[\left({\text{EC}}_{1}-{\text{EC}}_{0}\right)/\left({\text{EC}}_{2}-{\text{EC}}_{0}\right)\right]\times 100$$

### 2.5 Determination of oxidative stress marker and GABA content

Oxidative stress markers, such as malonaldehyde (MDA) and carbonyl content, were determined according to Sarkar et al. (2024).

GABA (γ-aminobutyric acid) content in the treated leaves was assessed using the methodology of Nayyar et al. (2014). Briefly, tissue (0.5 g) was homogenized in 6% trichloroacetic acid (TCA), followed by centrifugation at 12,000 × *g* for 15 min. The aliquot was collected and mixed with diethyl ether (250 mL), vigorously shaken for 10 min, and centrifuged at 10,000×*g* for 10 min. The resulting aliquot from each tube was carefully removed and exposed to a stream of air for 30 min to ensure complete evaporation of ether. The ‘GABase’ assay was used to monitor the reduction of NADP to NADPH using a UV-vis spectrophotometer (Shimadzu, Kyoto, Japan, UV-1900) at 340 nm, with GABA (Himedia, India) as the substrate.

### 2.6 Determination of ROS content, histochemical and fluorescence-specific detection

To determine H_2_O_2_ content, shoot tissue was homogenized in 1% (w/v) TCA solution, followed by centrifugation at 12,000×*g* for 15 min under cold conditions, and the aliquot was preserved. It was then mixed with 5 mM potassium iodide in 10 mM phosphate buffer (PBS; pH 7.0), and absorbance was measured at 390 nm against a standard 30% (v/v) H_2_O_2_ (Velikova et al., 2000). Similarly, superoxide (O_2_^•^-^^) content was assessed using shoot tissue extracted in 50 mM PBS (pH 7.5). After centrifugation at 12,000×*g* for 15 min under cold conditions, the aliquot was collected and vigorously mixed with a reaction solution (60 mM PBS, pH 7.6, and 10 mM hydroxylamine) at 25°C for 12 min. The reaction was then halted using 10 mM sulfanilamide and 7 mM α-naphthylamine, and O_2_•^-^ content was measured spectrophotometrically at 530 nm using potassium nitrite as the standard (Das et al., 2024a).

Histochemical localization of ROS (H_2_O_2_ and O_2_^•^-^^) was performed using 3´,3’ diaminobenzidine (DAB) and nitroblue tetrazolium (NBT) staining, respectively. Briefly, treated leaf sheaths were dipped into the respective staining solution at room temperature for 12 h, followed by treatment with a dechlorophyllization solution under heated conditions. Once the pigments were cleared, the spots were captured using a digital camera for documentation.

The methodology described by Sarkar et al. (2024) was employed to detect tissue-specific H_2_O_2_ localization. Roots (~1 cm) were washed with ddH_2_O to remove debris, immersed in staining solution (20 μM 2´,7´- dichloroflourescein acetate; DCFH-DA) for 15 min, washed with PBS, mounted, and analyzed for fluorescence using a confocal microscope (Carl Zeiss LSM 800) with λ_ex_ 490 and λ_em_ 520 nm.

### 2.7 Analysis of pigment components, thermal images and Hill activity

Leaf pigments, such as chlorophyll, were estimated from 0.5 g of fresh leaves crushed in 80% (v/v) chilled methanol and centrifuged at 10,000 × *g* for 12 min. The resulting supernatant was collected, and absorbance was recorded at 652 and 665 nm using a UV–Vis spectrophotometer (UV-1900, Shimadzu, Kyoto, Japan), following the method described by Sarkar et al. (2023).


Total chlorophyll=22.12 A652+2.71 A665

Similarly, anthocyanin content was estimated according to the protocol of Mancinelli et al. (1975) with slight modifications. Fresh samples (0.5 g) were crushed and filtered in 10 mL of acidified methanol (1:99, HCl:methanol, v/v) using a mortar and pestle, incubated at 4°C for 24 h, and the volume was adjusted to 5 mL. After incubation, the homogenates were centrifuged at 5,000 × *g* (4°C) for 15 min. The resulting supernatant was preserved in 10 mL tubes at 25°C, and absorbance of the leaf extract was measured at 530 and 657 nm against a blank, using a molar extinction coefficient of 29,600 L mol^-^¹ cm^-^¹ (equivalent to cyanidin-3-glucoside).

To monitor thermal load in the rice canopy, infrared (IR) imaging was performed using a Fluke TiX580 Infrared Camera. The camera, securely mounted on a tripod and calibrated for emissivity, captured comprehensive thermal images of the canopy. Image processing and temperature computation were conducted according to the manufacturer’s protocol (Kaya et al., 2024).

The activity of photosystem II was estimated by measuring the reduction of 2,6-dichlorophenolindophenol (DCPIP) following 2 h of illumination of intact leaves. Tissues were homogenized in cold 50 mM Tris-HCl buffer (pH 7.8), filtered through Whatman 42 paper, and mixed with a sorbitol-containing buffer. After centrifugation at 10,000 × *g* (4°C, 12 min), the pellet was resuspended and stored on chilled Falcon tubes. For the assay, a chloroplast suspension (10 mg mL^-^¹ chlorophyll) was added to a reaction buffer containing 0.5 mM DCPIP and exposed to light (1000–1200 μE m^-2^ s^-1^) for 5 min, after which absorbance was recorded at 580 nm. Controls lacking chloroplasts or light were included. Hill activity was calculated using the formula described by Sarkar et al. (2023) and expressed as μg DCPIP reduced mg^-^¹ chl h^-^¹.

### 2.8 Quantification of PAs metabolism contents and enzymes activities

To determine endogenous polyamines (PAs), 0.5 g of plant tissue from each treatment was crushed in 10% (v/v) perchloric acid using a mortar and pestle, followed by dansylation and separation on a thin-layer chromatography (TLC) plate (silica gel 60 F254, 20 × 20 cm) developed in ethyl acetate:acetone (3:1, v/v). Putrescine (PUT), spermidine (SPD), and spermine (SPM) were used as standards and quantified spectrophotometrically (Bouchereau et al., 2000).

For arginine decarboxylase (ADC) and ornithine decarboxylase (ODC) activity, 0.5 g of fresh leaf tissue was ground in 100 mM phosphate-buffered saline (PBS, pH 8.0) containing 0.1 mM phenylmethylsulfonyl fluoride (PMSF), 1 mM pyridoxal phosphate (PLP), 6 mM EDTA, 20 mM ascorbic acid, and 0.1% (w/v) polyvinylpyrrolidone. The homogenate was centrifuged at 12,000 × *g* at 4°C for 20 min. The supernatant was collected and incubated overnight at 4°C in darkness with 3 mL of 100 mM PBS containing 0.04 mM PLP, 0.2 mM DTT, and 0.3 mM EDTA. Absorbance was recorded at 340 nm (Piao et al., 2022).

Following Rodríguez et al. (2009), 0.5 g of fresh shoots was crushed in liquid nitrogen and homogenized in 1.5 mL of 100 mM PBS (pH 6.5). The homogenate was centrifuged (12,000 × *g*, 4°C, 20 min), and the resulting supernatant was collected. It was then incubated in a reaction mixture containing 100 μM 4-aminoantipyrine (4-AAP), 1 mM 3,5-dichloro-2-hydroxybenzenesulfonic acid (DCHBS), 0.06 mg/mL horseradish peroxidase, and 100 mM PBS (pH 6.5) for 3 min at room temperature. The reaction was initiated by adding 7 μL of 10 mM SPD, and absorbance at 515 nm was measured over 1 min to calculate H_2_O_2_ generation. Using the Lineweaver–Burk method, the inhibition constant (*K_i_*) for SL-11061 was calculated for polyamine oxidase (PAO), yielding a *K_m_* of 17.7 μM. Diamine oxidase (DAO) activity was assessed following the methodology described by Scoccianti et al. (1990).

### 2.9 Transmission electron microscopy and scanning electron microscopy analysis

Leaf tissue sections (1 cm) were placed in Eppendorf tubes containing 4% (w/v) glutaraldehyde and 4% (w/v) paraformaldehyde in 50 mM sodium cacodylate buffer (pH 7.0). The samples were fixed overnight at 4°C and then washed in the same buffer for 4 h at 4°C. Subsequently, the samples were transferred to a TEM fixative [1% (w/v) osmium tetroxide] for 3 h, followed by rapid dehydration through a graded ethanol series (each step lasting 30 min). The dehydrated samples were embedded in Spurr’s resin, and 2 µm-thick sections were cut using an ultramicrotome, mounted on slides, stained with 4% uranyl acetate (12 min) and lead citrate (3 min), and examined at 5 kV using a transmission electron microscope (Tecnai G220 S-TWIN) to detect ultrastructural alterations.

For SEM analysis, plant tissues were washed with 100 mM phosphate-buffered saline (PBS, pH 7.5), fixed in 2.5% (v/v) glutaraldehyde, and dehydrated through a graded ethanol series (30 min each step). The samples were then analyzed using a scanning electron microscope (ZEISS EVO LS 10) (Das et al., 2025).

### 2.10 Visualization of cell death and callose formation in root tissue

To visualize root meristem cell death, root tips (~1 cm) from each treatment were stained with propidium iodide (PI; 5 mg/L) for 12 min in the dark. Excess stain was removed by washing, and the root samples were mounted on slides and observed under a confocal microscope equipped with a Z-stack function. Focused images were captured at excitation/emission wavelengths λ_ex_ 493 nm and λ_em_ 636 nm.

Callose deposition was visualized by aniline blue staining, following the method described by Piršelová et al. (2012).

### 2.11 Determination of H^+^-ATPase content and programmed cell death assay in root

Root-specific plasma membrane H^+^-ATPase activity was assessed following the methodology of Hejl and Koster (2004). For the programmed cell death (PCD) assay, root segments (0.5 cm) were immersed in 0.1% (w/v) Evans Blue solution prepared in 100 µM CaCl_2_ (pH 5.6) in test tubes. After 30 min of incubation, the samples were removed, rinsed with double-distilled water (ddH_2_O), and transferred to 3 mL of 70% (v/v) ethanol and 1% (w/v) sodium dodecyl sulfate to solubilize dead cells. Absorbance was measured at 600 and 680 nm using a UV–Vis spectrophotometer (Chen et al., 2011).

### 2.12 Antioxidant defense system

Antioxidant enzyme activity was assessed by homogenizing 0.5 g of fresh leaf tissue in an extraction buffer containing 100 mM phosphate-buffered saline (PBS, pH 6.8), 0.3 mM EDTA, 1 mM phenylmethanesulfonyl fluoride, and 1% polyvinylpyrrolidone (Campos et al., 2019). The homogenate was centrifuged at 12,000 × *g* for 15 min at 4°C, and the supernatant was used to measure the activities of superoxide dismutase (SOD), peroxidase (POD), and catalase (CAT). SOD activity was determined following the method of Shukla et al (2023), POD activity was assessed using the protocol of Das et al (2024b), and CAT activity was measured according to the methodology described by Tewari et al. (2013).

### 2.13 Gene expression analysis

To analyze gene expression using real-time quantitative PCR (qRT-PCR), total RNA was extracted using a commercial RNA extraction kit and reverse-transcribed into cDNA with a specific cDNA synthesis kit (GCC BIOTECH, India). qRT-PCR was performed on a BIORAD CFX™ system using SYBR Green Master Mix (TaKaRa Biomedicals, Japan). Gene expression levels of ethylene signaling genes were calculated using the 2^–ΔΔCt^ method with primers designed from the NCBI database (**Supplementary Table S1**; Wang et al., 2023). *Ubiquitin 5* (*UBQ5*) was used as the internal reference gene. All qRT-PCR assays were conducted with three biological replicates.

### 2.14 Statistical analysis

Data represent the means ± standard error (SE) from three independent biological replicates per treatment. Statistical significance was determined using one-way analysis of variance (ANOVA) followed by Tukey’s *post hoc* test for multiple comparisons. Differences at *p* ≤ *0.05* were considered statistically significant and are indicated by different alphabetic letters.

## 3 Results

### 3.1 Optophysical analysis of PUT-nZnO nanoentities

**Figure 1:**
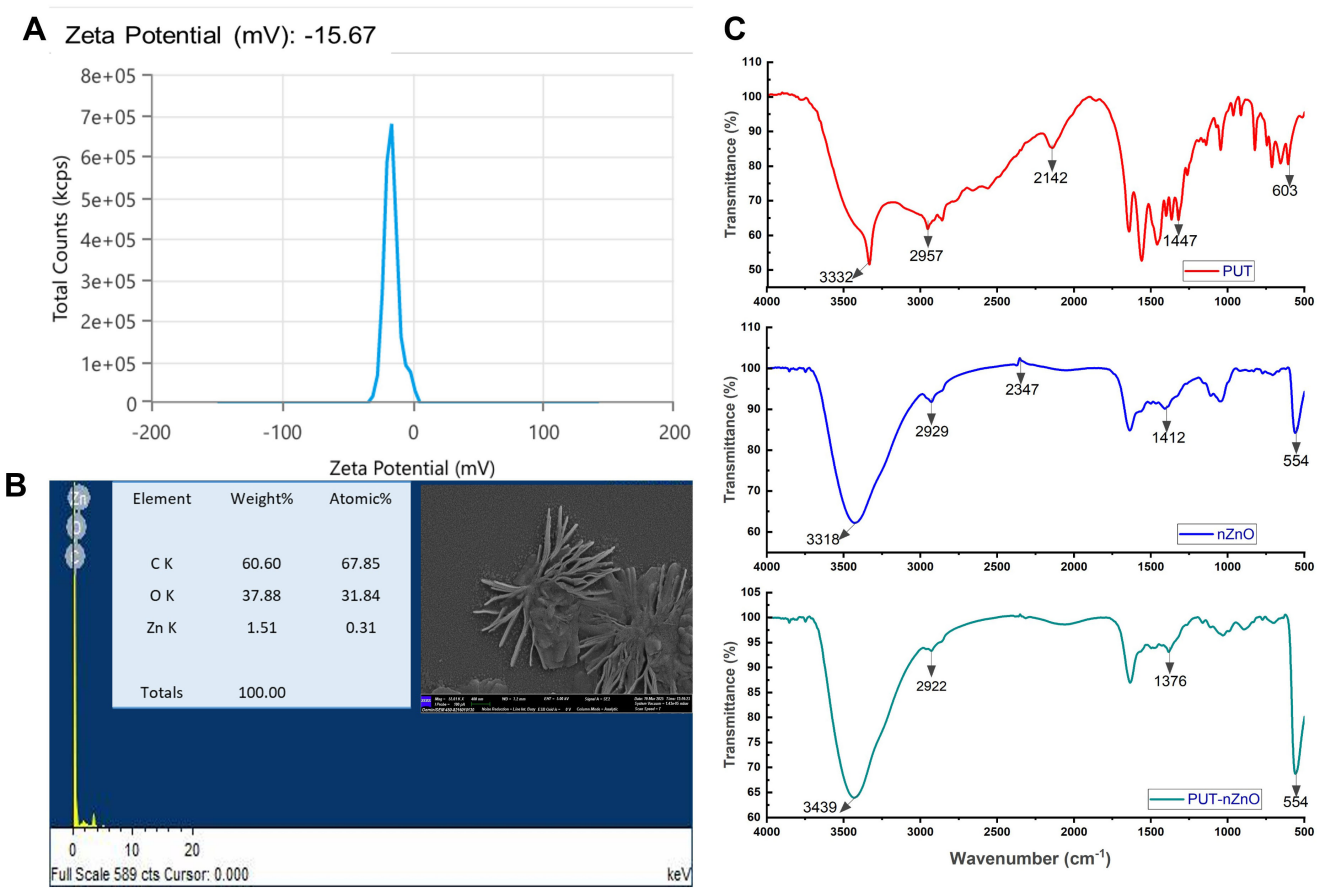
Characterization of PUT-nZnO. **(A)** Zeta potential, **(B)** SEM image of PUT-nZnO nanoentities with elemental mapping through EDX-analysis and **(C)** FTIR-imaging of respective compounds with shareable functional-bonds.

The biofabricated nZnO surface was modified using putrescine (PUT), a biometabolite, during the synthesis of the nanoentities. The PUT-nZnO nanoentities were characterized using various nano-characterization techniques (**Figure 1**). Zeta potential analysis revealed a metallic core with hydrodynamic stability; the negative value (−15.76 mV) indicates colloidal stability and surface modification through the polycationic nature of polyamines (PAs). Field emission scanning electron microscopy (FE-SEM) analysis showed that PUT-nZnO exhibited a star-shaped morphology, while elemental mapping confirmed the presence of abundant Zn in the nanoentities. Fourier-transform infrared (FTIR) spectroscopy validated the conjugation of PUT with nZnO by identifying functional bonding interactions. Notable peaks included: 3439 cm^-1^ [O-H stretching vibration; hydrogen bonding interactions between PUT and nZnO], 2957 cm^-1^ [C-H stretching vibrations; alkanes or methyl (-CH_3_) and methylene (-CH_2_-) groups], 2929 cm^-1^ [C-H stretching vibrations; methylene (-CH_2_-) groups], 1376 cm^-1^ [C-H bending vibrations in methyl (-CH_3_); involved in symmetric deformation of CH_3_ groups], and 554 cm^-1^ [metal-oxygen (M-O) stretching vibrations], which characterize the molecular interactions responsible for the bioactivity of PUT-nZnO.

### 3.2 Impact of drought stress on growth attributes and membrane stability

**Figure 2:**
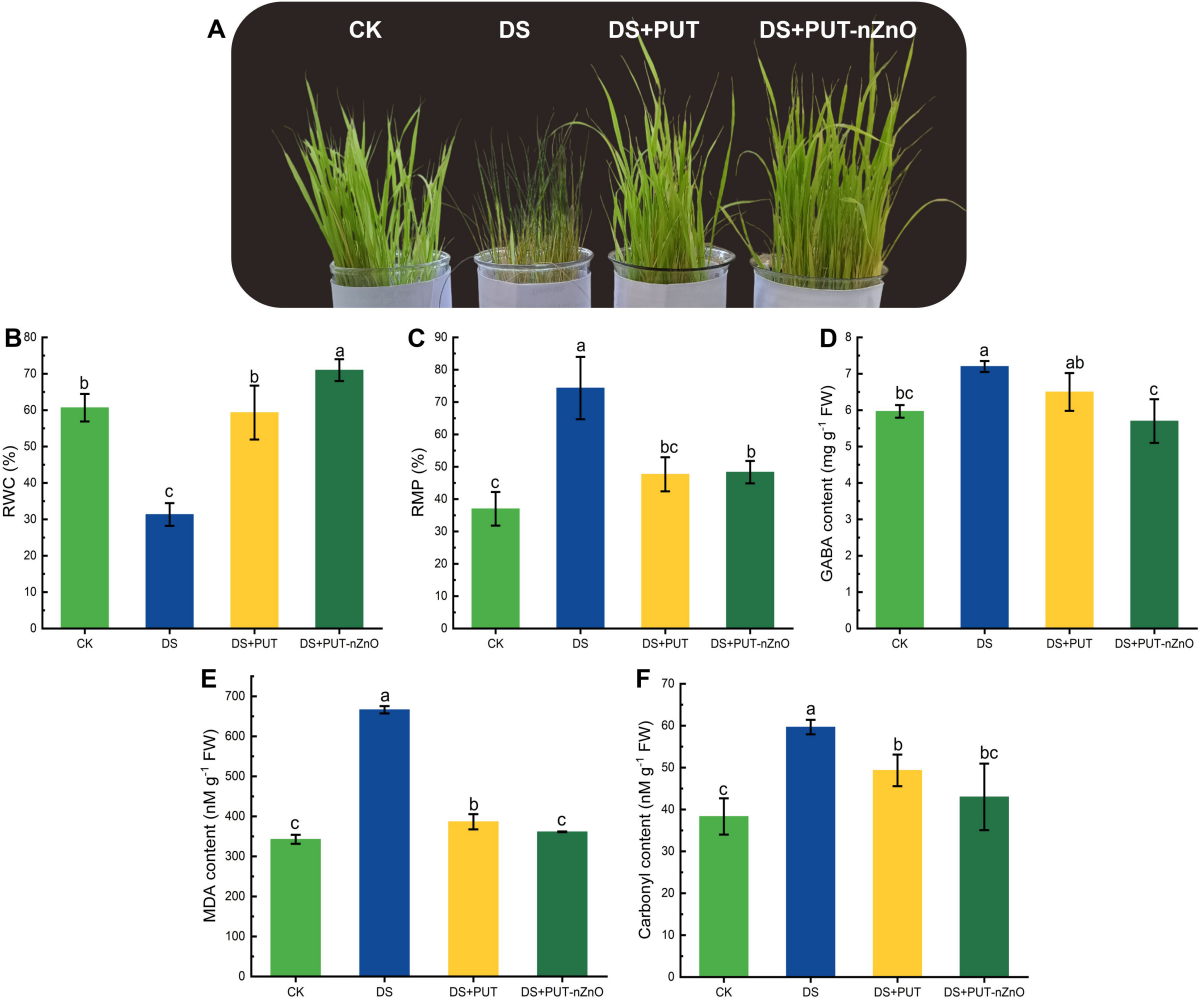
Images showing changes of **(A)** plant phenotypes, **(B)** RWC, **(C)** RMP, **(D)** osmoregulator like GABA, key oxidative marker – **(E)** MDA and **(F)** carbonyl content of rice seedlings subjected to different treatments under 12% PEG-induced DS for 14-D over CK. Data expressed as means ± SE (n = 3). Different letters indicate significant differences (ANOVA, *p≤ 0.05*).

Exposure to 12% PEG-induced DS significantly (*p* ≤ *0.05*) affected the growth of rice seedlings (cv. Swarna Sub1), triggering oxidative stress. However, this stress was effectively alleviated through foliar application with PUT or PUT-nZnO (**Figure 2A**). Under DS, relative water content (RWC) was substantially reduced. Foliar application (prior to DS induction) of PUT and PUT-nZnO maintained tissue hydration levels at 89% and 126%, respectively, compared to DS alone (**Figure 2B**). Similarly, relative membrane permeability (RMP) exhibited the same trend under 14-day PEG-induced DS with respective treatments (**Figure 2C**).

The accumulation of membrane peroxidation markers significantly (*p* ≤ *0.05*) damaged root tissues. Malondialdehyde (MDA) and protein carbonyl content increased by 94% and 55% under DS relative to the control (CK), whereas PUT-nZnO application reduced MDA and carbonyl accumulation by 45% and 27%, respectively (**Figures 2E, F**). DS also elevated γ-aminobutyric acid (GABA) levels, indicating enhanced metabolic stress (**Figure 2D**). Foliar application of PUT-nZnO restored GABA levels by 20%, approaching CK levels. These results suggest that nZnO acts synergistically with PUT to enhance plant resilience against drought-induced oxidative stress.

### 3.3 Induction of ROS and antioxidative response under stress

**Figure 3:**
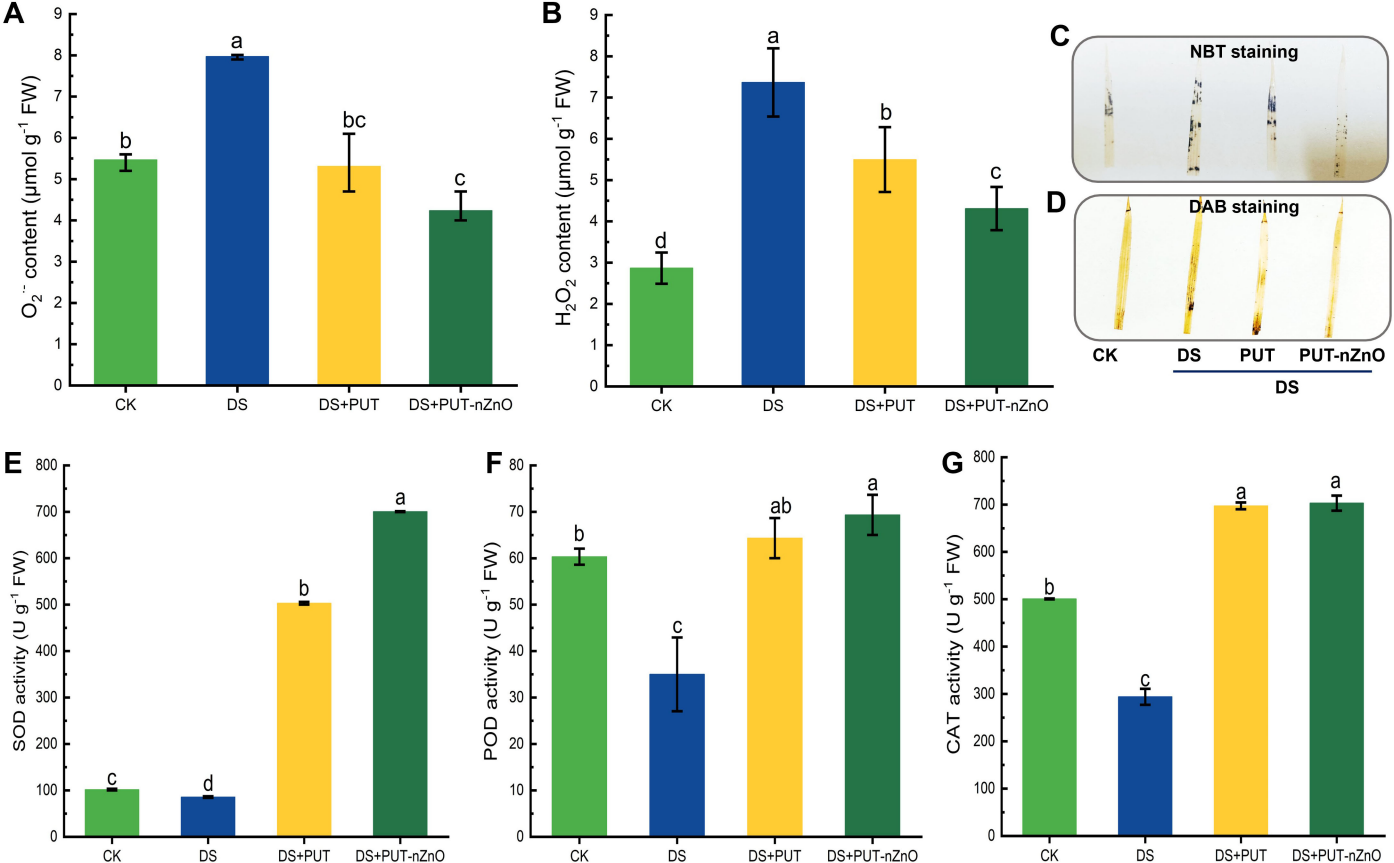
DS-induced oxidative damage in rice leaves. Spectrophotometric estimation of O_2_^•^-^^ and H_2_O_2_ **(A, B)**, respectively and histochemical visualization of respective ROS molecules through **(C)** NBT and **(D)** DAB staining on leaf lamellae. Changes in major antioxidants **(E)** SOD, **(F)** POD, and **(G)** CAT activity, subjected to 12% PEG-induced DS for 14-D over CK, with retrieval by DS+PUT and DS+PUT-nZnO. Data expressed as means ± SE (n = 3). Different letters indicate significant differences (ANOVA, *p≤ 0.05*).

Drought stress, alternatively transformed into a state of oxidative redox imbalance, was documented in rice seedlings through the significant accumulation of ROS despite treatments. Oxidative stress resulted in a significant (*p* ≤ *0.05*) accumulation of both O_2_•^-^ and H_2_O_2_ in a correlated manner, with DS increasing their content by 45% and 69%, respectively, compared to the CK (**Figures 3A, B**). The reduction in O_2_•^-^ and H_2_O_2_ content by 33% and 41% under DS+PUT and DS+PUT-nZnO treatments, respectively, indicates minimized oxidative damage.

We observed tissue-specific distribution and histochemical imaging of O_2_•^-^ and H_2_O_2_ generation *in vivo*, with distinct patches noted on the laminal surface. ROS distribution, as assessed through histochemical color reactions using NBT and DAB staining, showed reduced intensity under DS+PUT and DS+PUT-nZnO compared to DS alone (**Figures 3C, D**).

For enzymatic antioxidation, SOD—the first line of defense against oxidative stress—was suppressed by 15% under DS compared to CK (**Figure 3E**). However, plants exhibited a significant recovery, with SOD activity increasing by 487% and 717% under DS+PUT and DS+PUT-nZnO, respectively. A similar trend was observed for POD, where its activity was downregulated by 41% under DS compared to CK, but modulated by 83% and 98% under DS+PUT and DS+PUT-nZnO, respectively (**Figure 3F**).

A comparable pattern was noted for CAT, although no significant (*p* ≤ *0.05*) variation was observed between DS+PUT and DS+PUT-nZnO (**Figure 3G**). Still, CAT activity was upregulated by 137% and 139% under the respective treatments, contributing to the breakdown of H_2_O_2_ in tissues under the same conditions.

### 3.4 Drought induced heat stress, scanning micrograph for stomatal behavior and photosynthetic pigments

**Figure 4:**
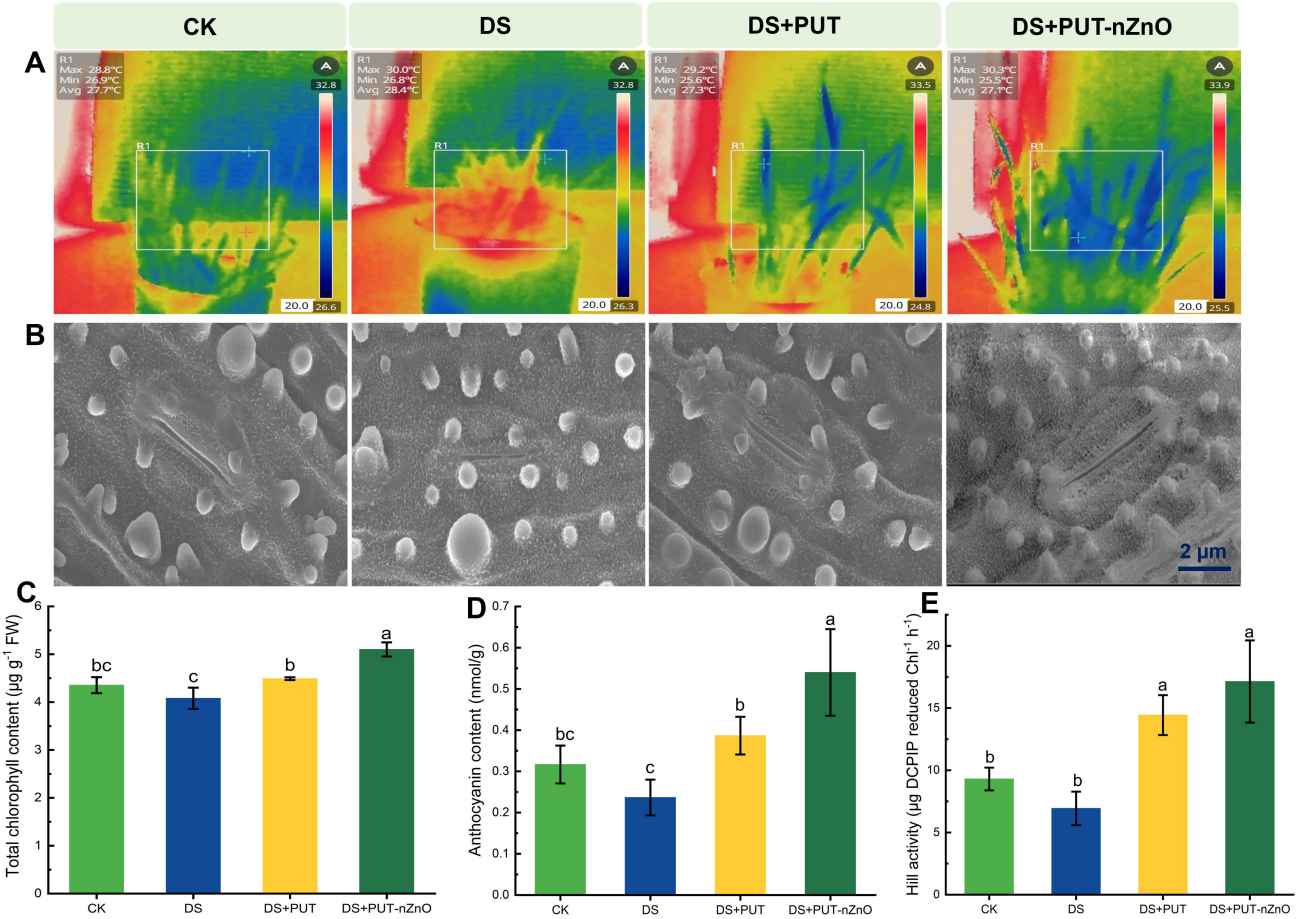
Effect of PUT-nZnO on photosynthetic regulation under drought-induced heat stress. **(A)** Infrared thermography showing canopy spatiotemporal variations, **(B)** scanning micrographs depicted behavior of stomatal aperture, **(C)** chlorophyll, **(D)** anthocyanin content, and **(E)** stability of PS-II through Hill activity on 14-D stressed rice leaves. Scale bars indicate the variations of temperatures. Data expressed as means ± SE (n = 3). Different letters indicate significant differences (ANOVA, *p≤ 0.05*).

Rice (cv. Swarna Sub1) seedlings were sensitized by DS, leading to stomatal closure (due to reduced transpirational cooling), as clearly evident in **Figure 4A**. Leaves exhibited an increased canopy temperature, with maximum energy emission observed under DS compared to the CK. Minimization of energy radiation, as sectorized within the boxed panel, resulted in changes in coloration. A similar trend was observed under DS+PUT compared to DS, suggesting that the leaf surface dissipated energy via long wavelengths.

However, stomatal conductance or transpiration rate was not measured in this study. Still, we predict the reopening of stomata under DS+PUT-nZnO treatment compared to DS, which is assumed to correlate with the RWC of leaves. Regulation of heat sensitization by leaf tissues may support chlorophyll biogenesis and related functions such as the Hill reaction. Plants retained significantly higher chlorophyll accumulation under DS+PUT, with levels 17% higher than under DS alone (**Figure 4C**).

Photochemical quenching, restored under PUT-nZnO treatment, was enhanced by 147% over DS, as indicated by Hill reaction activity (**Figure 4E**). While DS+PUT and DS+PUT-nZnO treatments did not differ significantly (*p* ≤ *0.05*) in Hill activity, they showed notable differences in anthocyanin accumulation (**Figure 4D**). The reduced anthocyanin level under DS (25% below CK) was restored by 68% and 134% under DS+PUT and DS+PUT-nZnO, respectively.

We observed that DS reduced water loss by affecting stomatal behavior (**Figure 4B**). Compared to CK, stomatal pores appeared smaller due to constriction under DS. However, turgidity and volume of guard cells and other subsidiary tissues were more normalized under DS+PUT and DS+PUT-nZnO treatments. Widening of stomatal pores under these treatments likely indicates improved tissue hydration. Notably, increased stomatal apertures and density under DS+PUT-nZnO contributed to maintaining leaf surface hydration and the native tissue structure, enabling the plant to resist dehydration.

### 3.5 Ultrastructural of chloroplast and real-time expression of *OsEREBP1* and *OsEGY1*

**Figure 5:**
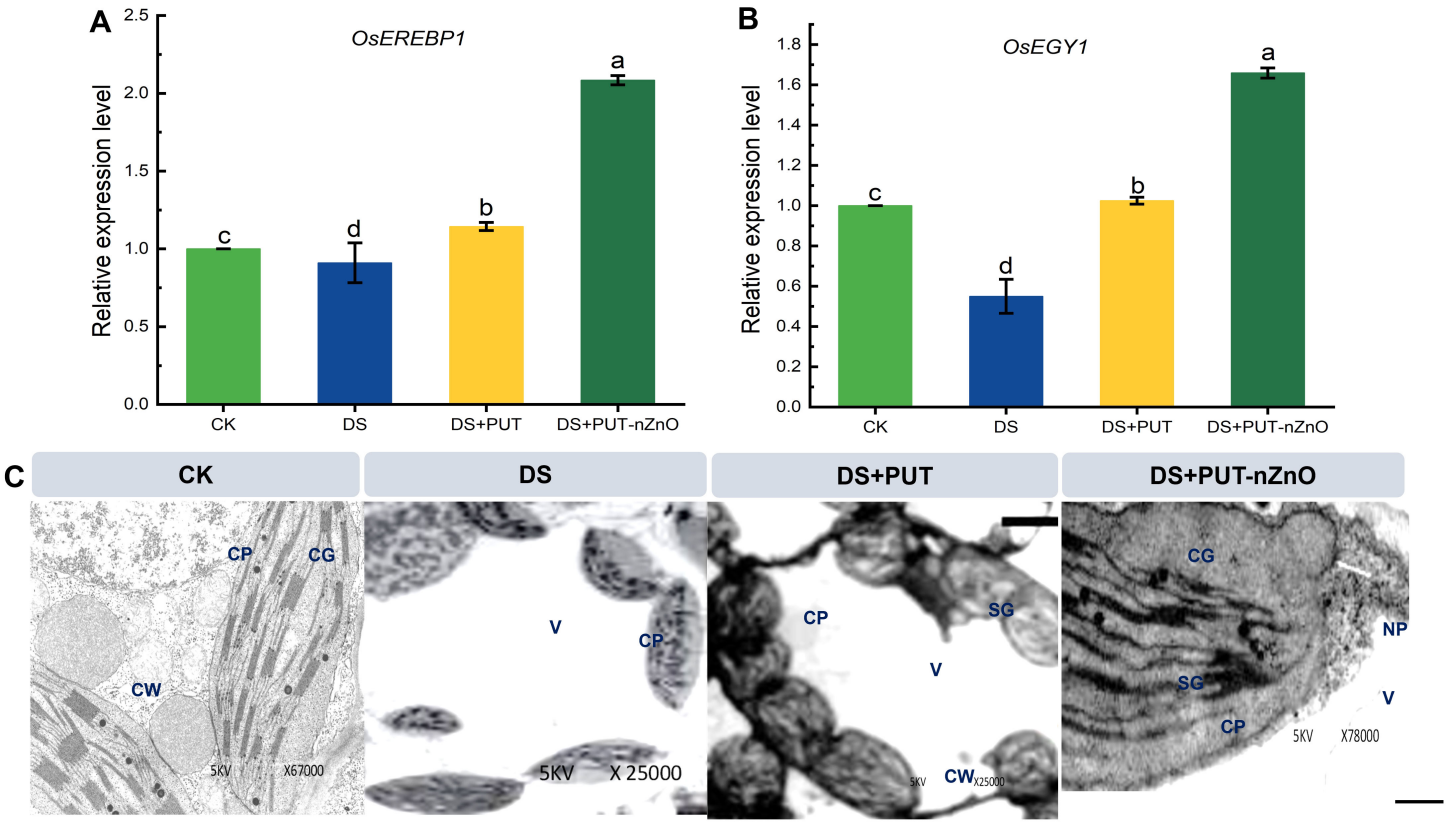
Relative expression level of ethylene signaling genes in rice leaves. mRNA expression of **(A)** *OsEREBP1* and **(B)** *OsEGY1* genes in rice leaves exposed to 12% PEG-induced DS for 14-D over CK. Data expressed as means ± SE (n = 3). Different letters indicate significant differences (ANOVA, *p≤ 0.05*). **(C)** Ethylene associated mRNA regulating chloroplast sustenance through TEM-microscopic imaging under 14-D drought-stressed rice leaves with different treatments. CW, cell wall; CP, chloroplast plastoglobuli; CG, chloroplast granum; V, vacuoles; SG, starch granule; NP, nZnO.

From the images obtained through TEM, we observed significant damage to the chloroplast membrane in rice seedlings under DS. Leaves subjected to 12% PEG-induced dehydration showed clear disorganization of the inner chloroplast membrane and stroma lamellae (**Figure 5C**). Loosening and collapse of thylakoid membranes indicated swelling or compression of the organelle, distinct under DS compared to CK. Starch-like granules were lightly distributed in the stroma, though their presence was not pronounced.

Treatment with PUT-nZnO mitigated structural damage, leading to more organized thylakoid stacking. While PUT alone supported structural retention, the combination treatment appeared more effective. The ultrastructure under DS may be associated with *OsEGY1* (Ethylene-dependent Gravitropism-deficient and Yellow-green 1) expression, which followed a recovery trend under both PUT and PUT-nZnO treatments. Chloroplast membrane stability was found to correlate with increased *OsEGY1* expression—upregulated by 98% and 221% compared to DS (**Figure 5B**).

Similarly, seedling sensitivity to ethylene-responsive gene modulation under DS was assessed via *OsEREBP1* (Ethylene Responsive Element Binding Protein 1) expression (**Figure 5A**). DS reduced its expression by 18% relative to CK, but this was reversed by PUT-nZnO. A steeper upregulation of *OsEREBP1* (106%) under PUT-nZnO characterized the treatment as more effective in ethylene metabolism, possibly due to the compatibility of ethylene and PA pathways within the same biosynthetic route.

### 3.6 DS triggers PAs metabolic activity

**Figure 6:**
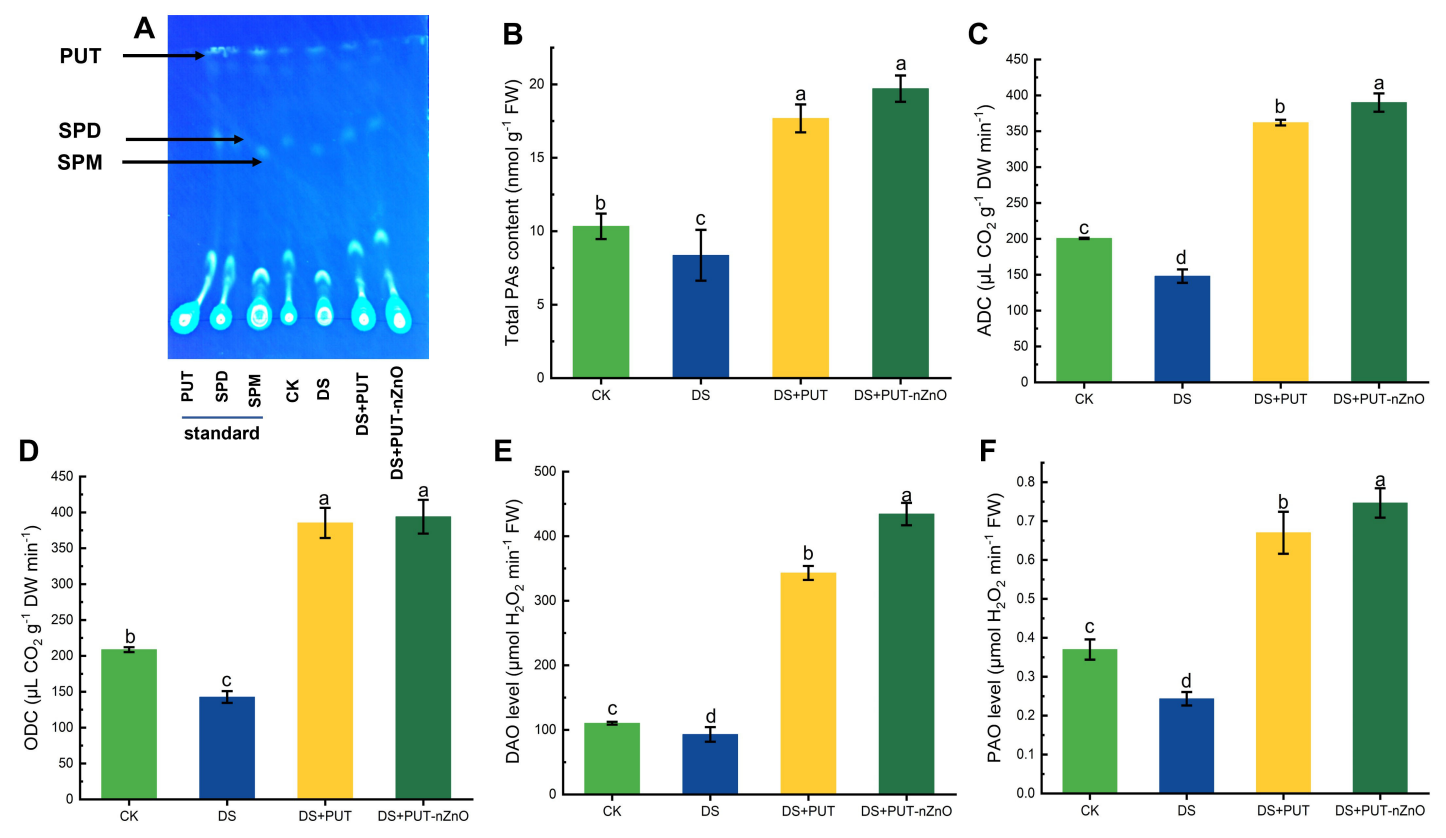
DS activates PA metabolic activity. **(A)** Changes in total PA levels analyzed via TLC methodology, **(B)** quantification of total PAs, and activities of **(C)** ADC, **(D)** ODC, as well as assays of **(E)** DAO and **(F)** PAO in rice leaves exposed to 12% PEG-induced DS for 14-D over CK, with retrieval by DS+PUT and DS+PUT-nZnO. Data expressed as means ± SE (n = 3). Different letters indicate significant differences (ANOVA, *p≤ 0.05*).

Polyamine (PA) accumulation and metabolism were found to be significantly responsive in rice seedlings, as indicated by changes in both biosynthetic and catabolic enzyme activities. Initially, PAs extracted in a suitable solvent were subjected to chromatographic separation using a fluorescence dye and quantified by a spectrofluorimetric method. The separation of PA fractions against standard solutions (PUT, SPD, SPM) produced distinct major fluorescent spots on the chromatogram, along with a few minor spots (**Figure 6A**).

The total PA concentration (bound + free) in leaf tissues increased by 19% under DS compared to the CK. Plants significantly upregulated PA levels (*p* ≤ *0.05*), with increases of 111% and 135% under DS+PUT and DS+PUT-nZnO treatments, respectively (**Figure 6B**). Similarly, PA biosynthetic pathways were assessed through the *in vitro* activity of ADC and ODC, showing a direct correlation with total PA content (**Figures 6C, D**). ADC activity was suppressed by 26% under DS but recovered by 144% and 163% under DS+PUT and DS+PUT-nZnO, respectively, reflecting the treatments’ stimulatory effect on key biosynthetic pathways.

ODC, a rate-limiting enzyme in an alternative PA biosynthetic route, followed a similar pattern. Its activity declined by 46% under DS but was upregulated by 171% and 177% under DS+PUT and DS+PUT-nZnO, respectively.

To evaluate PA catabolism, *in vitro* activity of DAO and PAO was assayed (**Figures 6E, F**). Enzyme activities associated with PA catabolism followed a similar trend, with maximum induction observed under DS+PUT-nZnO. Specifically, DAO and PAO activities increased by 268% and 211%, respectively, suggesting enhanced PA oxidation that contributes to cellular homeostasis under stress.

### 3.7 Mortality of tissues and membrane activity

**Figure 7:**
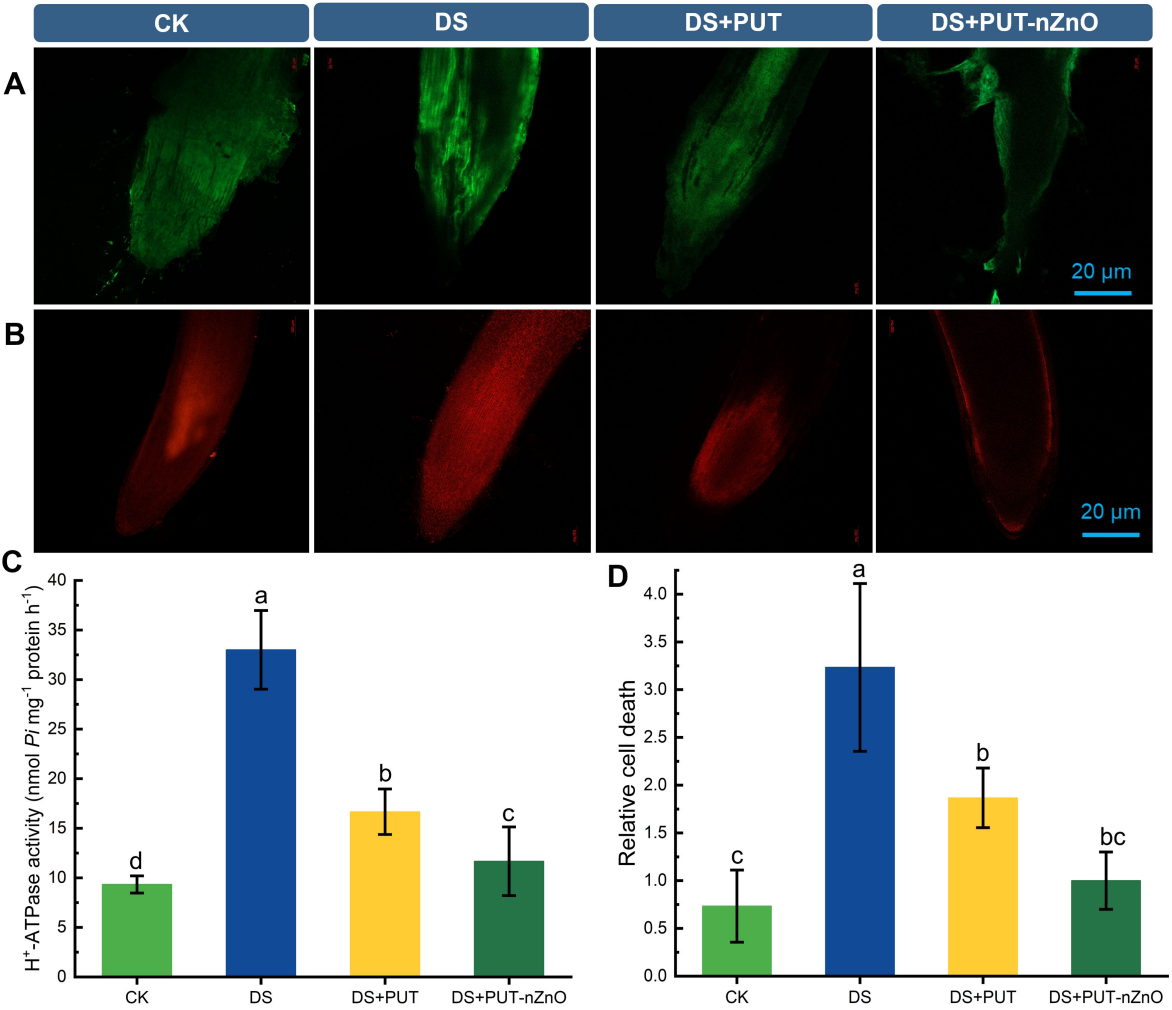
Visualization of fluorescent probe specific ROS and PCD in rice roots under DS. ROS **(A)** DCF-DA staining for ROS labelling and **(B)** PI-based fluorescence images of PCD in root tips of rice seedlings. **(C)** Spectrophotometric estimation of H^+^-ATPase and **(D)** relative cell death of rice roots subjected to 12% PEG-induced DS for 14-D over CK. Data expressed as means ± SE (n = 3). Different letters indicate significant differences (ANOVA, *p≤ 0.05*).

Loss of cell viability in tissues—particularly due to exposure to ROS coupled with dehydration—was validated using DCF-DA and PI sensitivity-based imaging (**Figures 7A, B**). Root tissues showed significant vulnerability, with cell lysis accompanied by elevated ROS levels and oxidative damage. PI staining revealed degeneration, with fluorescence extending from the cortex into the central core under DS. However, DS+PUT and DS+PUT-nZnO treatments restricted PI diffusion to the epidermal and hypodermal layers, effectively limiting tissue degeneration.

Similarly, increased DCF-DA fluorescence under DS indicated DNA damage through cortical layers. Reduced fluorescence under DS+PUT-nZnO treatment suggests improved resistance to ROS-induced lysis, likely due to protective roles of PAs and/or nZnO, with damage restricted to the epidermis.

Relative PCD resulting from ROS-induced lipid peroxidation was assessed spectrophotometrically using Evans Blue staining of roots under DS. PA and nZnO treatments appeared to suppress lipoxygenase activity and protect membranes from ROS attack, leading to more diffused Evans Blue staining. DS+PUT-nZnO treatment was especially effective in reducing damage at root tips and the meristematic region, helping preserve overall root structure (**Figure 7D**).

Physiological viability was further assessed by monitoring ion transport via H^+^-ATPase activity in rice seedling roots (**Figure 7C**). Anticipating ion loss under DS, H^+^-ATPase activity was upregulated by 253% compared to CK, indicating a compensatory mechanism to restore ionic balance. To further examine membrane transport sensitivity, H^+^-ATPase activity was tested with KCl (inducer) and sodium orthovanadate (Na_34_;; inhibitor) (data not shown). Adjustment of ionic balance was achieved through a significant downregulation of enzyme activity by 49% and 64% under DS+PUT and DS+PUT-nZnO treatments, respectively.

### 3.8 Variations in root and leaf surface morphology and associated callose deposition

To validate our experimental results on drought sensitivity over 7 days and the impact of PUT-nZnO, root surface morphology was examined. Significant (*p* ≤ *0.05*) changes were observed in the epidermis under DS, including tissue disorganization, downstream progression of damage, scant development of bristle-like structures, and altered surface morphology compared to the well-hydrated CK.

Initial signs of DS sensitivity included loosening of epiblema tissues due to cell wall dissolution. Moisture deficiency led to constriction of ground tissue cell volume, causing longitudinal furrow formation on the root surface. The emergence of appendage-like root caps further indicated drought-induced structural responses.

**Figure 8:**
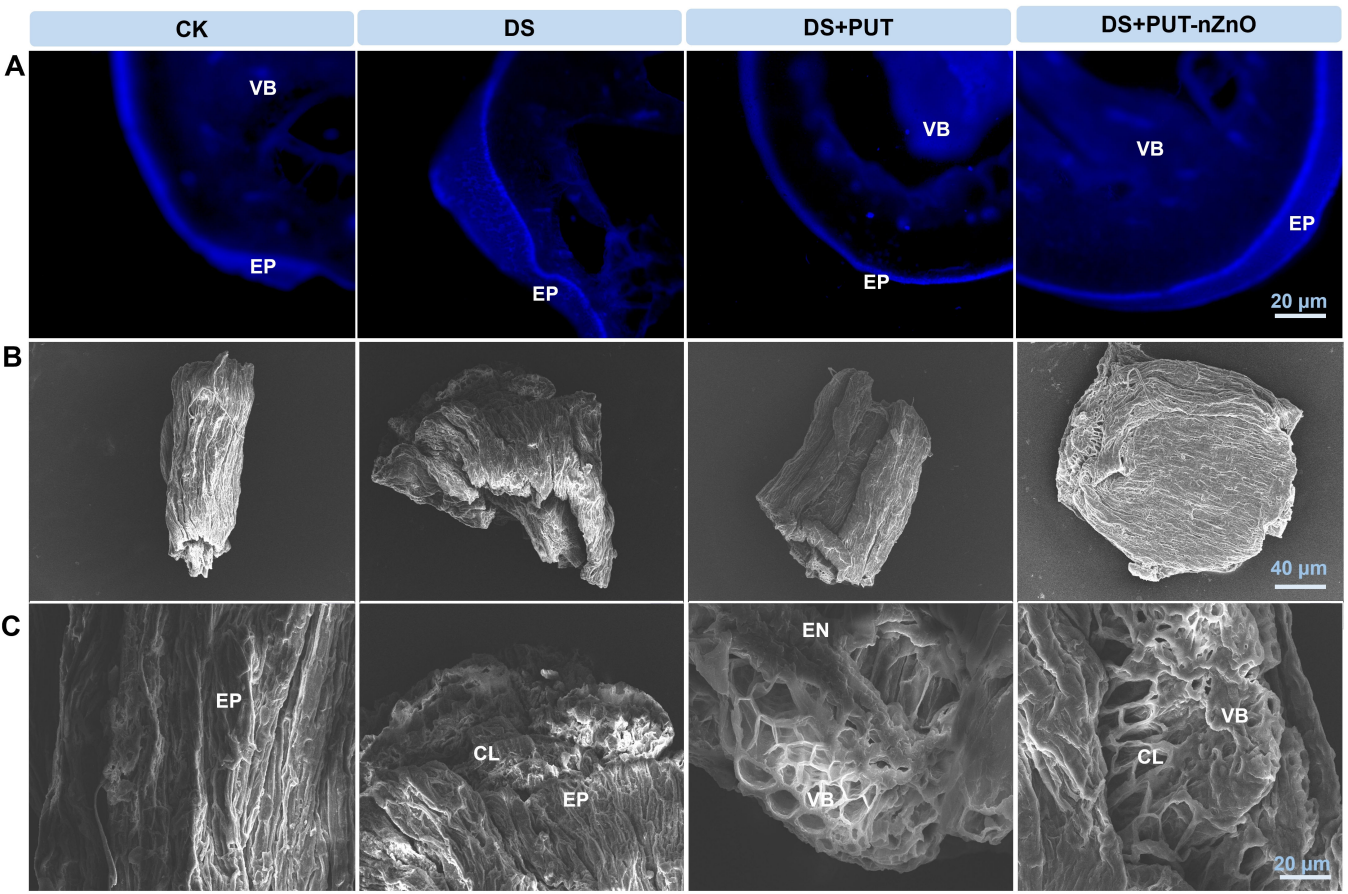
**(A)** Visualization of fluorescent probe specific callose labeling in roots under 12% PEG-induced DS for 14-D over CK, with retrieval by DS+PUT and DS+PUT-nZnO. **(B)** Scanning electron micrographs showing topography on the surface of drought-stressed rice roots under various treatments. **(C)** Enlarged microscopic view highlights the inner root surface of drought-stressed rice under different treatments with retrieval by DS+PUT and DS+PUT-nZnO. EP, epidermis; CL, cortical layer; EN, endodermis; VB, vascular bundle.

However, exogenous application of PUT-nZnO markedly reduced epidermal cracking and helped preserve the native structure of the root cell wall (**Figures 8A, B**). Root metabolic activity under DS was altered, promoting the formation of complex polysaccharides such as callose (**Figure 8C**). DS sensitivity was associated with increased callose deposition, which may help prevent water vapor loss from endodermal cells. nZnO, either alone or in combination with PUT, enhanced callose synthesis in a more condensed form, contributing to drought tolerance.

Variations in the intensity of callose deposition were observed across treatments, as visualized by UV fluorescence. Callose was primarily localized to the protoxylem and bundle sheath, with pronounced accumulation in both DS and DS+PUT-nZnO treatments. Specific sites with intense callose staining exhibited noticeable cell wall thickening and elevated fluorescence intensity.

### 3.9 Correlation among different treatments and parameters

**Figure 9:**
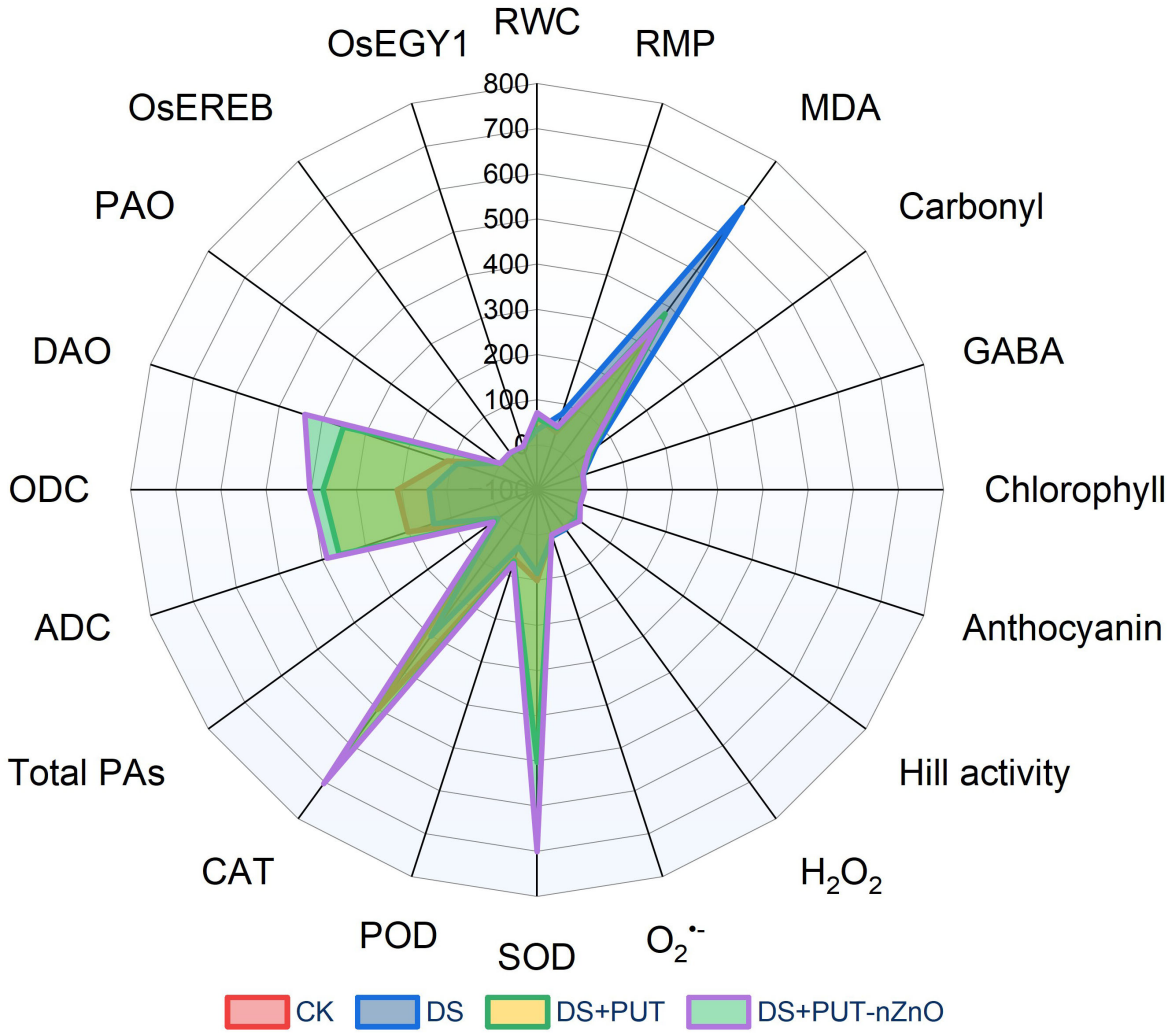
Radar plot summarizes the interactions between major cellular and molecular attributes in response to different treatments of DS-exposed rice seedlings.

The radar plot (**Figure 9**) clearly illustrates how PEG-induced DS negatively impacts key biochemical modules, such as oxidative balance and the plant–water relationship. Under DS (blue coloration in the graph), key oxidative markers—MDA and carbonyl content—are elevated, while a noticeable decrease in RWC indicates tissue dehydration.

The addition of PUT (DS+PUT; green) provides partial relief by modulating chlorophyll and PA metabolism. Remarkably, the combined application of nanoentities (DS+PUT-nZnO; purple coloration) shows striking improvements in plant resilience under 14-day drought exposure. This treatment not only restores tissue hydration but also enhances photosynthetic efficiency and strengthens antioxidant defenses, leading to substantial mitigation of ROS.

Moreover, the plot suggests that ethylene-signaling activity (*OsEGY1* and *OsEREBP1*) may reflect molecular-level regulation that contributes to alleviating drought-induced damage. In summary, this analysis opens new paths to explore how nZnO, when combined with PUT, can synergistically strengthen plant defense mechanisms at both physiological and molecular levels.

## 4 Discussion

To understand the regulatory mechanisms of DS, a number of hypotheses and experimental findings have established two principal pathways: DS-induced osmotic adjustment and the maintenance of cellular redox balance in dehydrated tissues (Parisa et al., 2025). In rice seedlings, drought sensitivity primarily affects root growth and solute acquisition, which in turn negatively regulates shoot development and root branching and restricts leaf area, among other outcomes (**Supplementary Figure S1**). Several other factors have also been shown to support drought tolerance, either individually or in combination with other cellular responses.

We induced DS through PEG-mediated tissue dehydration for a defined period to observe drought-sensitizing responses and their modulation by PUT-nZnO. While individual applications of PUT or nZnO have previously been reported to enhance drought tolerance, their combined use may represent a novel formulation. Exogenous chemical elicitation has proven effective in promoting osmoprotectant accumulation, reducing membrane denaturation, limiting oxidative degradation of biomolecules, enhancing growth-related signaling, and activating antioxidant cascades.

In anticipation of the effectiveness of biocompatible substances in enhancing drought tolerance in rice, we formulated PUT-nZnO and observed significant alterations in cellular and nuclear activity in treated seedlings. Interestingly, the rice genotype used in this study, cv. Swarna Sub1, has been reported to carry regulatory elements within the QTL *Sub1A*, which is associated with drought sensitivity. *Sub1A*, an ethylene-responsive factor (ERF), is believed to mediate ethylene signaling and interact with DS-sensitive cellular processes in plants (Adak et al., 2023).

Our findings are consistent with those of Khan et al. (2019), who reported that nZnO enhanced ethylene signaling in rice. Therefore, the combined application of PUT-nZnO is expected to enhance ethylene-mediated drought signaling pathways while simultaneously mitigating DS-induced oxidative damage.

### 4.1 Drought compromises both water relations and the oxidative status of tissues

Primarily, DS is a critical limiting factor in rice cultivation, causing impaired root growth and disrupting both physiological and metabolic functions. In our experiment, PEG-mediated dehydration—measured at approximately −0.54 MPa—proved highly detrimental, significantly suppressing seedling growth compared to the control group. At the cellular level, DS results in substantial moisture loss, though not to the extent of permanent wilting. This moisture deficit impairs key membrane functions, especially ion and solute permeability (**Figure 2C**).

The application of PUT-nZnO effectively sustained RWC and reduced RMP, thereby contributing to the restoration of membrane integrity and functionality. Ionic imbalance driven by DS becomes more pronounced when roots lose ions along concentration gradients—particularly potassium (K^+^), a key element for maintaining cellular turgor and membrane potential. This imbalance extends to mesophyll tissues in leaves. Stabilizing the diffusion pressure deficit across vascular conduits requires the active acquisition of K^+^ ions against their concentration gradient, a key mechanism underlying drought tolerance (Feng et al., 2016).

In our experiment, rice seedlings exhibited an upregulation of H^+^-ATPase activity specifically in response to PUT-nZnO treatment. This phenomenon may be attributed to PAs**-**mediated recovery of cellular hydration, primarily through the regulation of water channels (aquaporins) and ion channels that preserve membrane fluidity (Feng et al., 2016). Cellular osmotic adjustment under DS is more effective in rice genotypes possessing the *Sub1A* QTL—such as cv. Swarna Sub1 used in this study—which responded significantly to nZnO supplementation under moisture-deficit conditions. This response is likely regulated by *Sub1A*-mediated ethylene metabolism, which interacts with abscisic acid (ABA) to restore root hydraulic conductivity under dehydration stress (Das et al., 2024a).

Thus, it is plausible that PUT supplementation enhances the efficacy of nZnO by sustaining osmotic potential and promoting cellular viability under DS. This synergy was evident in the recovery of RWC and regulation of RMP, both of which were mirrored in improved downstream growth parameters, ensuring seedling survival. DS signaling is primarily initiated through changes in membrane dynamics, where specific secondary messengers play critical roles. In our study, the overproduction of GABA in response to PUT-nZnO treatment effectively restored osmotic turgidity in seedlings, strongly correlating with cellular hydration (**Figure 2D**). GABA, a well-established signaling molecule, can also promote osmotic balance by interacting with osmoprotectants and compatible solutes such as proline and glycine betaine. Therefore, the integration of PUT-nZnO appears promising for regulating DS signaling and tissue osmotic balance.

Water stress, frequently accompanied by oxidative stress, becomes especially severe under stomatal closure, rendering plants more vulnerable. Accordingly, DS-induced peroxidation of biomolecules—particularly lipids and carbonyl groups—remains a major concern. PAs have been widely reported to mitigate oxidative damage by shielding nucleophilic polymer backbones and stabilizing macromolecules (Agostinelli et al., 2010). Additionally, PAs are known to modulate oxidative stress by regulating key enzymes such as NADPH oxidase and xanthine oxidase. This outcome is supported by our measurements of ROS (O_2_^•^-^^ and H_2_O_2_) in DS-induced tissues, as well as their histochemical localization.

Collectively, the application of PUT-nZnO nanoentities appears highly effective in controlling oxidative stress—an inevitable component of DS in rice.

### 4.2 Polyamine content and its catabolism regulate oxidative stress during plant-water deficit

Plants synthesize polyamines (PAs) from a common precursor, methionine, via a divergent pathway that also leads to ethylene biosynthesis. Under stress conditions—particularly drought-induced stomatal closure—osmotic regulation is disrupted, promoting abscission and lodging through ethylene signaling. However, this response can be mitigated by PA interference, which plays a critical role in stabilizing root membrane integrity, enabling efficient water uptake, and regulating oxidative stress under DS.

Compared to previous reports, drought-tolerant genotypes with higher endogenous PA levels exhibit enhanced resistance to osmotic stress, partly due to their influence on the accumulation of proline-like metabolites (Kavi Kishor et al., 2022). PA metabolism is critically linked to its catabolism, which generates reactive oxygen species (ROS) such as H_2_O_2_. While H_2_O_2_ is considered a safer, biocompatible ROS, it must remain within threshold concentrations to prevent toxicity. At optimal levels, H_2_O_2_ acts as a signaling molecule, activating antioxidative enzymes and other antioxidants (Yadav et al., 2025). Therefore, PAs indirectly aid in ROS scavenging, reducing oxidative stress and preventing membrane degradation. In some cases, PAs themselves have been reported to function as antioxidants, supporting the ascorbate–glutathione pathway by neutralizing superoxide and hydroxyl radicals. In this way, they also contribute to H_2_O_2_ regulation and enhance cellular resilience against ROS invasion (Anam et al., 2024).

This is further illustrated by the increased PA content observed in rice seedlings treated with PUT, which exceeded the synergistic effect of PUT-nZnO (**Figure 6A**). PUT, a key precursor of higher-order PAs, is known to stimulate the synthesis of additional PA derivatives, effectively enlarging the total PA pool. In our study, we also observed differential accumulation of PA fractions—such as SPM and SPD—as well as other unidentified residues. However, the collective PA content was significantly upregulated in treated tissues compared to CK. The role of PAs in maintaining water balance is largely attributed to their ability to preserve membrane integrity through electrostatic interactions with negatively charged domains, such as those regulated by *OsEREBP1* expression of membrane-associated aquaporins. PA-mediated modulation of channel proteins further facilitates ion transport across membranes—particularly for Zn—and this interaction appears to correlate with total PA content (Khan et al., 2019).

In this context, PUT-nZnO nanoentities, in which Zn is incorporated in nanoform, likely exhibit distinct modalities for PA biosynthesis and signaling. The accumulation of PAs, whether in free or conjugated forms, strongly correlates with drought tolerance by regulating physiological traits such as membrane permeability and root hydraulic conductivity (Das et al., 2024a). Therefore, nZnO successfully complements PUT in increasing total PA content, as demonstrated in our experiment, to promote downstream stress tolerance mechanisms.

PUT-mediated antioxidation is evident in tissues through the accumulation of ROS (e.g., H_2_O_2_), which in turn activates antioxidative pathways. While H_2_O_2_ is not a free radical, it is generated in plant tissues by copper-containing peroxidases and amine oxidases that utilize PAs as substrates. The involvement of PAs in H_2_O_2_ formation is beneficial for maintaining low, controlled concentrations that initiate key intracellular signaling processes, including ion transport, homeostasis regulation, and oxidative redox sensing (Gupta et al., 2016).

Beyond its role in photorespiratory C_2_ cycling, PAO-mediated H_2_O_2_ accumulation contributes to oxidative stress regulation via sulfur residue formation. Additionally, H_2_O_2_-mediated signaling from PA hydrolysis may enhance the activity of major antioxidants, such as glutathione—particularly when co-supplemented with selenium nanoparticles (Das et al., 2025). Our experiment supports this, showing upregulation of DAO and PAO activity upon exposure to nanomaterials such as nZnO, which effectively modulate oxidative stress while also improving Zn nutrition.

Notably, drought-induced programmed cell death (PCD) or senescence—often exacerbated by excessive ROS production—is mitigated by PAs through both direct scavenging and activation of antioxidative enzymes (Panda et al., 2024). This protective effect is further enhanced when nZnO provides additional shielding against ROS-mediated cell damage under DS. Therefore, beyond their individual roles, the combined application of PUT and nZnO appears to be more effective in supporting plant resilience against DS-induced cellular damage.

### 4.3 Sustained energy conversion into organic residues supports drought tolerance in plants

Earlier studies on the conversion of solar energy into chemical energy through photosynthetic carbon reduction pathways (PCRP) have identified this process as an important strategy for enhancing drought tolerance (Reddy et al., 2004). Rice, being a semi-aquatic plant, is particularly vulnerable to carbon loss through respiratory burn-off, photosynthetic carbon oxidation, and photorespiration. Soil moisture deficit and its intrusion into mesophyll tissues through vascular conduits lead to stomatal closure, restricting CO_2_ concentration and consequently curtailing photosynthetic carbon reduction. This issue is further exacerbated under intense illumination exceeding the light compensation point in C_3_ plants (e.g., rice), where photooxidation of tissues occurs.

In our experiment, rice seedlings exhibited stabilized photosystem-II (PS-II) activity, indicating reduced photosynthetic quenching, as measured by Hill activity (**Figure 4E**). We hypothesize that PUT molecules, either alone or in combination with nZnO, effectively mitigate PS-II degradation by preserving membrane-bound electron transfer proteins and the oxygen-evolving complex. Additionally, PUT-induced expression of chloroplast proteins helps maintain the native structure of PSII, with *OsEGY1* sustaining Hill activity (Jahan et al., 2022). Thus, the PUT-nZnO nanoentities used in our experiment could serve as a reliable anti-senescence regulator by detoxifying ROS, enhancing antioxidative pathways (SOD, POD, and CAT, herein), and promoting broad-spectrum tolerance to sustain chloroplastic integrity and function. This conclusion is supported by ultrastructural observations of chloroplasts from different treatment groups, which revealed severe disorganization of the stroma lamellae. DS also induces thylakoid membrane constriction, reducing lumen volume and compromising chloroplast structure in rice (Wang et al., 2019). Under DS conditions, chloroplasts appear discrete, suspended in a disordered cytosol, with the disappearance of other organelles. Similar findings have consistently aligned with changes in photosynthetic ultrastructure under DS, which were reversed through the application of PUT.

Under DS, with stomata closed, rice leaves exposed to high irradiation experience thermal stress. Significant changes in canopy temperature in rice fallows serve as indicators of plant-water stress. Evaporation acts as a natural mechanism for heat dissipation, releasing long-wavelength radiation to counteract thermal stress. Consequently, thermal imaging in our study presents a non-destructive approach for evaluating rice seedlings, where PUT-nZnO treatments proved effective. Energy dissipation captured by thermal imaging indicates reduced DS severity, confirming the beneficial role of nZnO (Kareem et al., 2022). Thus, the ability to harness energy for photochemical reactions while dissipating thermal load under DS is a critical factor in regulating plant function. Our findings suggest that PUT, whether applied independently or in combination with nZnO, significantly contributes to this process.

### 4.4 Ethylene-mediated senescence: a key factor in drought sensitivity and its regulation by PUT-nZnO

Dehydrated roots exhibit distinct collapse, with membrane permeability significantly affected, leading to electrolyte leakage. In rice seedlings, specifically in the cv. Swarna Sub1 genotype, we recorded membrane stabilization under 12% PEG (−0.57 MPa)-mediated DS, demonstrating tolerance through regulated EL. In the present study, PUT-nZnO effectively contributed to membrane structure restoration, achieving maximum reduction in EL. The role of PAs in shielding membranes with negatively charged residues facilitates stress avoidance under dehydrated soil conditions (Gupta et al., 2016). Additionally, nZnO can supplement Zn as a nutrient to dehydrated root tissues while also co-transporting K^+^ across membranes to maintain stable osmotic turgidity. Subsequently, the minimization of EL through PUT-nZnO applications has been recognized as a reliable approach for promoting early drought tolerance in rice seedlings.

Remarkably, PAs, in collaboration with other volatile growth regulators such as ethylene, are interconnected through a common pathway that plays a pivotal role in ensuring normal plant development. They regulate a multitude of physiological, biochemical, and molecular processes associated with growth and adaptation under both optimal and stressful environmental conditions. Therefore, the synergism among these molecules often plays a crucial role in shaping plant responses to environmental fluctuations (Jangra et al., 2023). Furthermore, premature tissue abscission under DS is associated with the hydrolytic activity of root walls, where ethylene interference plays a significant role. Ethylene biosynthesis and its transport to dehydrated tissues trigger the transcription of multiple genes within a specific regulatory system. Genes induced by ethylene interact with regulatory sequences and bind to specific transcription factors, co-expressing other genes involved in drought sensitization. Ethylene-responsive binding proteins (ERBP), a class of AP2/ERF proteins, exhibit homology with regulators of other genes, including ABA (Feng et al., 2020).

Ethylene and PAs, as two important growth regulators in plants, share a common biosynthetic pathway where arginine and methionine are key precursors. The biosynthesis of ethylene is also regulated by PAs. Interference with chloroplast senescence under dehydration is linked to ethylene accumulation and the induction of senescence-activated genes (Xing et al., 2024). At the cellular level, PAs counter ethylene signaling by interacting with ethylene receptors and modulating downstream signaling components. PUT acts as an inhibitor of ACC synthase, the rate-limiting enzyme in ethylene biosynthesis. This membrane-associated enzyme may be influenced by PUT, from which ethylene signaling is initiated (Tabur et al., 2024). In our study, we predict that the downregulation of ethylene signaling by PUT in combination with nZnO may facilitate chloroplast survival and prevent induced senescence under DS.

Moreover, previous literature suggests that overexpression of *ERBP* enhances root permeability, promotes stomatal closure, and facilitates the development of dehydrin and late embryogenesis abundant (LEA) proteins, with *OsEGY1* modulating physiological and cellular functions to support drought tolerance. In rice seedlings, *OsERBP* expression also alters chloroplast ultrastructure, influencing photosynthetic energy conservation and contributing to partial drought tolerance (Khan et al., 2021). Our experiment observed upregulated expression of *OsERBP* in response to PUT-nZnO, suggesting potential ethylene signaling interference for drought tolerance. Drought avoidance mechanisms—including stomatal closure, gravitropic root movement, callose formation, and root meristem differentiation—are closely linked to ethylene signaling. Moreover, ethylene metabolism communicates with PA metabolism, where PA oxidation controls ROS generation, activating further defense mechanisms against DS.

The sustenance of chloroplasts and the integrity of thylakoid ultrastructure are crucial for CO_2_ and water vapor transport through stomata under DS. Stress-induced protective proteins, such as metalloproteases, operate independently of ATP-mediated binding within thylakoids. Additionally, the expression of the *EGY1* gene, encoding a ~59 kDa metalloprotease, is frequently reported in *Arabidopsis*, where it supports chloroplast development under water stress and stabilizes chlorophyll a/b-binding proteins in light-harvesting complex II (Sanjaya et al., 2021). In our study, we also measured chlorophyll content (25%), which evaluated the response of cv. Swarna Sub1 to PUT-nZnO under stress conditions (**Figure 4C**). Additionally, we observed an abundance of transcripts, suggesting a role in maintaining chloroplast structure and function. *OsEGY1* expression provides additional advantages for DS tolerance, as the chloroplast stroma houses key Calvin cycle enzymes such as RuBisCO, which must maintain their native structure. Drought stress-induced stomatal closure creates an alkaline pH (>8.0), potentially causing denaturation of carbon-concentrating enzymes and impairing their optimal activity. This exacerbates insufficient CO_2_ reduction under limited intracellular CO_2_ concentration, leading to excess photo-generated ATP and subsequent photooxidation. As a metalloprotease, *OsEGY1* is capable of degrading unwanted polypeptides and has been correlated with ethylene accumulation under DS. Although ethylene accumulation can lead to premature chloroplast senescence or abscission, *OsEGY1* provides protective effects through its metalloprotease activity (Luciński and Adamiec, 2023). Thus, while the full array of PUT-nZnO interactions within cellular systems is yet to be fully explored, our findings suggest that this formulation could effectively safeguard the native integrity of chloroplasts, mitigating DS-induced injury.

### 4.5 Cell viability in roots: a key criterion for early sensitivity and drought tolerance

The viability of tissues that reflect plant tolerance under DS is collectively manifested through various cellular functions. Under DS, the loss of viability is initiated in root meristematic tissues, where specific dyes can penetrate only the damaged membranes in comparison to the untreated ones. Our observation of intense PI coloration under DS, relative to the control, directly indicates the loss of membrane integrity in degenerating tissues. PUT-nZnO proved most effective in controlling membrane function loss by minimizing PI inclusion.

Furthermore, PI staining is also indicative of the genotoxicity induced by DS, as the dye binds to DNA released from the nucleus through damaged cell membranes. In rice, an inverse relationship has been reported between PI-mediated fluorescence intensity and cell viability in roots under combined drought and salinity stress (Mira et al., 2017). Given the inherent nature of PUT to bind nucleophilically with DNA, nZnO exhibits strong potential in resisting nuclear damage and enhancing tolerance to genotoxicity. Additionally, as a micronutrient, Zn—particularly in its nanostructured form—helps repel harsh metal ions from the cell membrane, reducing mechanical injuries. Moreover, the localization of DCF-DA in root cells confirms the role of ROS in the breakdown of cortical cell membranes. Changes in cortical structure and nuclear DNA damage are also recognized as key indicators of PCD (Wany et al., 2017).

In this study, rice seedling roots subjected to DS exhibited cytoplasmic DNA aggregation due to epidermal wall fragmentation, followed by cytoplasmic collapse and root distortion. This correlated with root tip cell death, indicating that high ROS levels induced PCD, contributing to nuclear DNA damage, organelle degradation, and membrane collapse in meristematic cells. These findings suggest that PUT-nZnO nanoentities shield root plasma membranes through PUT’s polycationic nature and nZnO’s water retention capacity. The results suggest that corpse clearance efficiently removes dead cells. This process is regulated, especially in the lateral root region, where cells undergo continuous replacement and PCD as part of a coordinated mechanism under severe water deficit.

### 4.6 PUT-nZnO enhances drought sensitivity: a modulation of root adaptation and callose-mediated osmoregulation

Under DS, the root surface morphology of rice seedlings in this study distinctly revealed features related to intracellular structure and stomatal abundance. As plants underwent 14 days of DS exposure, the most significant physiological response was the minimization of stomatal apertures. Cellular hydration in epidermal tissues—particularly guard and subsidiary cells—facilitated stomatal reopening under PUT-nZnO treatment. ZnO, both in nanoform and when combined with chitosan, demonstrated a strong ability to rescue tissue hydration through the synthesis of compatible solutes that generate a more negative water potential (Inam et al., 2024). Additionally, epidermal cell roughness and cutinization—marked by stomatal regulation—function as adaptive traits for minimizing energy loss and reducing the vapor pressure deficit outside the stomata under DS. This essential drought-avoidance feature is common in many cereals and is further enhanced by chemical treatments acting as osmoregulators.

In the present case, rice seedlings may have gained improved access to mechanisms that reduce evapotranspiration via microscopic furrows on the root epidermis, particularly when exogenous PUT was applied. This phenomenon also highlights the electrostatic binding of PAs to oppositely charged residues in the cell wall, effectively preventing stress-induced injuries such as ion leakage and gas bubble entry into vascular conduits. The latter becomes particularly concerning under DS, as cavitation formation disrupts hydrostatic pressure within xylem water columns.

In seedlings, root meristem growth is impaired by ionic effects in a water-deficit environment. In the present case, roots of cv. Swarna Sub1 subjected to DS showed more collapsed endodermal layers and cavity shrinkage (**Figures 8A,B**). This is corroborated by stele distribution through apoplastic spaces, where osmotic stress–induced conjugated PAs likely play an important role in modulating membrane integrity and boosting H^+^-ATPase activity. This activity supports water retention and nutrient uptake, where nZnO acts as a carrier.

Overall, the PUT-nZnO formulation demonstrated significant drought-mitigating effects in this study. Drought avoidance in cereals is also physiologically manifested through the development of specific hydrophobic polysaccharide residues within the cell wall, with callose formation playing a crucial role. Chemically, callose is a β-1,3-glucan—an excretory product of the cell wall—that reduces vapor pressure deficits under critical rhizosphere moisture conditions (Das et al., 2025). Callose formation is often facilitated through ABA biosynthesis in roots, which simultaneously enhances osmotic concentration through compatible solutes within the root epidermis.

Thus, nZnO-mediated recovery of cellular hydration under soil-moisture deficit is assumed to be linked to callose development, as previously documented (Nandhini et al., 2019). However, the correlation between ZnO concentration (whether in nano or bulk form) and callose deposition may vary across genotypes. In fact, callose deposition is a dynamic physiological adaptation regulated by both synthetic (callose synthase) and hydrolytic (β-1,3-glucanase) processes, with minimal direct correlation to Zn absorption from the soil. Nonetheless, the involvement of nZnO in callose formation is likely indirect, with ABA serving as the primary modulator for drought avoidance strategies.

## 5 Conclusion

In essence, DS appears as a key inducer in the development of oxidative stress through the overproduction of ROS, leading to susceptibility in rice seedlings. This study revealed the recovery of drought tolerance through the application of PUT, nZnO, or their combination. Primarily, drought tolerance is achieved by PAs protecting cell membranes and nZnO modulating cellular pathways. When PUT is complexed with nZnO, significant improvements in root–shoot biomass, RWC, regulation of EL, and development of GABA—all of which support dehydration tolerance—were observed.

The ameliorative pathways of the PUT–nZnO composite are facilitated by the following mechanisms:.

1. efficient ROS scavenging through antioxidation,
2. biosynthesis of osmolytes
3. increased PA pool and its oxidation to release H_2_O_2_ for signaling,
4. preservation of chloroplast structure and associated ethylene signaling, and
5. maintenance of root plasma membrane integrity, thereby reducing DNA loss.

These pathways constitute a possible coordinated strategy to combat DS through neutralization of harmful ROS with improved antioxidant ratios, compensation of cellular fluidity via ionic balance, development of signaling residues such as PAs and H_2_O_2_ transcriptional activation of chloroplast-protective genes, and sustained membrane integrity in roots.

**Figure 10:**
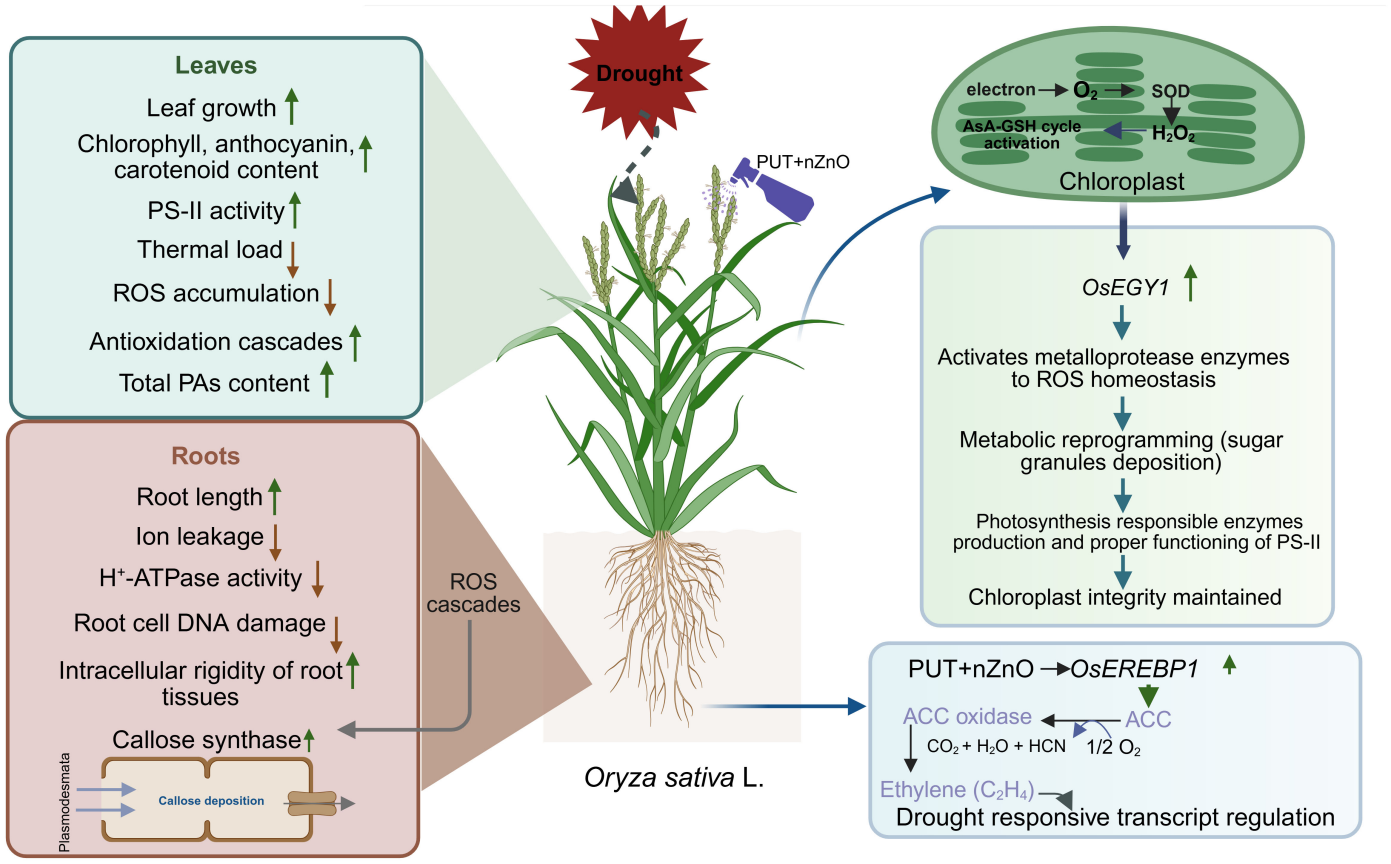
Diagram of rice plants responses to 12% PEG-induced DS through foliar application of PUT-nZnO nanoentities.

Therefore, the combinational effects of PUT and nZnO add significant value to rice seedlings, primarily due to the protective nature of PAs against dehydration injuries and the enhancement of specific physiological responses by nZnO, collectively boosting drought tolerance (**Figure 10**). This study also underscores the compatibility of nanoparticles (e.g., nZnO) with organic residues (PAs), which jointly trigger regulatory mechanisms to sustain plant growth under DS—although concerns regarding phytotoxicity remain unresolved.

Mechanistically, this tolerance reflects enhanced cellular hydration and membrane stability, along with the recovery of redox homeostasis disrupted by oxidative stress. The present findings highlight the potential of metallic nanoparticles biofabricated with plant-derived residues to augment stress resilience.

Contextually, the PUT–nZnO combination may represent a novel formulation capable of improving drought resilience in rice seedlings, though its current validation is confined to *Sub1A* QTL-bearing cultivars. Other genotypes remain untested. This formulation effectively mitigated DS-induced osmotic perturbation and secondary oxidative damage, with PUT conferring ROS protection and nZnO mediating downstream antioxidant signaling.

These effects were physiologically substantiated through enhanced biomass accumulation, improved RWC, and GABA accumulation—key indicators of dehydration tolerance. The formulation also supported ROS detoxification, organelle integrity, PA biosynthesis, H_2_O_2_-mediated signaling, and ion retention, reinforcing its role in DS mitigation.

Therefore, the synergistic efficacy of PUT and nZnO warrants field-level validation. The PUT–nZnO complex may serve as a promising biocompatible agent, meriting further investigation under field conditions, where DS varies diurnally. Its mode of action could also be dissected using omics-based approaches to gain mechanistic insights. In future, this innovative composition is expected to undergo translational field trials for enhancing early seedling resilience in rice in a biocompatible manner while adapting to other environmental factors. 

## Data Availability

The raw data supporting the conclusions of this article will be made available by the authors, without undue reservation.
