# Supplementary material for: Exploring regulatory roles of putrescine-doped zinc oxide nanoentities on ethylene signaling, redox imbalance, and programmed cell death in drought-stressed rice (Oryza sativa L.) seedlings
Source: Front Plant Sci. 2025 Aug 19;16:1630837. doi: 10.3389/fpls.2025.1630837 (PMC12401919; doi:10.3389/fpls.2025.1630837)
Supplement: Supplementary file 1 [file DataSheet1.docx]

**Supplementary information**

**Exploring regulatory roles of putrescine-doped zinc oxide nanoentities on ethylene signaling, redox imbalance, and cell death in drought-stressed rice (*Oryza sativa* L.) seedlings**

Abir Das^1^, Tibor Janda^2^*, Sudipta Kumar Sil^3^, Malay Kumar Adak^1^*

*^1^Plant Physiology and Molecular Biology Research Unit, Department of Botany, University of Kalyani, Kalyani, West Bengal 741235, India*

*^2^Department of Plant Physiology and Metabolomics, Agricultural Institute, HUN-REN Centre for Agricultural Research, Martonvasar 2462, Hungary*

*^3^Department of Botany, University of Gour Banga, Malda, West Bengal 732103, India*

*Corresponding Email: [janda.tibor@atk.hun-ren.hu](mailto:janda.tibor@atk.hun-ren.hu) (TJ); [malay009@klyuniv.ac.in](mailto:malay009@klyuniv.ac.in) (MKA)


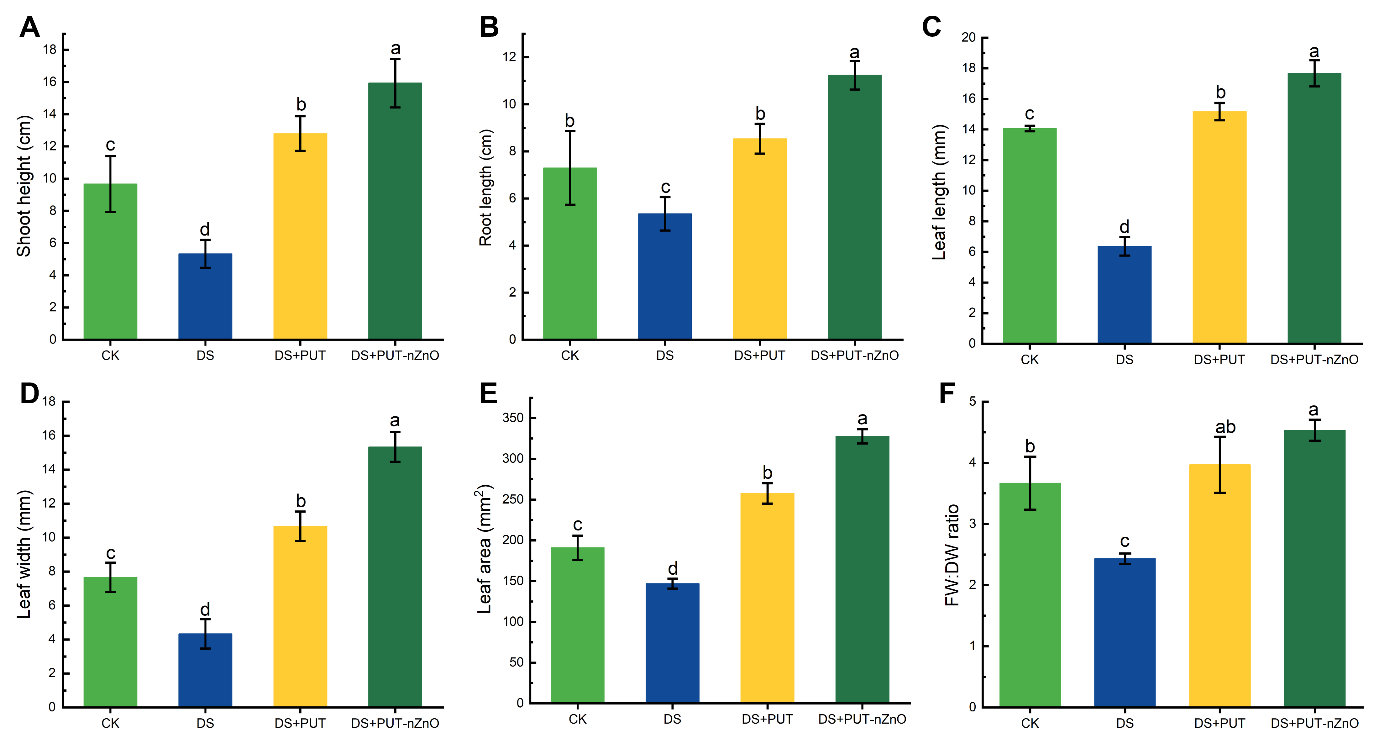


**Figure S1.** Changes in plant growth attributes (A) shoot height, (B) root length, (C) leaf length, (D) leaf width, (E) leaf area, and (F) ratio between fresh weight (FW) and dry weight (DW) of plant biomass of rice seedlings subjected to different treatments under 12% PEG-induced DS for 14-D over CK. Data expressed as means±SE (n = 3). Different letters indicate significant differences (ANOVA, *p≤ 0.05*).

**Table S1** Oligonucleotide primer sequences used for RT-qPCR analysis

| Gene name | Primer sequences (5´ 3´)  Forward sequence Reverse sequence | |
| --- | --- | --- |
| *Ubiqutin5 (UbiQ5)* | ACCACTTCGACCGCCACTACT | ACGCCTAAGCCTGCTGGTT |
| *OsEREBP1* | ACTGCCGGATTTGATGGTCCTG | CAGCATCATAAGCTCTTGCAGC |
| *OsEGY1* | CCGGTGGCTTATGGTGTCTT | TTCGGATGCACAACTGCTCT |
